# Phylogenetic signal of sub‐arctic beetle communities

**DOI:** 10.1002/ece3.8520

**Published:** 2022-02-16

**Authors:** Samantha E. Majoros, Sarah J. Adamowicz

**Affiliations:** ^1^ Department of Integrative Biology University of Guelph Guelph ON Canada

**Keywords:** Arctic, biogeography, DNA barcoding, entomology, environmental filtering, macroecology, phylogenetic community structure

## Abstract

Postglacial dispersal and colonization processes have shaped community patterns in sub‐Arctic regions such as Churchill, Manitoba, and Canada. This study investigates evolutionary community structure within the beetle (Coleoptera) families of Churchill and tests whether biological traits have played a role in governing colonization patterns from refugial and southerly geographic regions. This study quantifies sub‐Arctic beetle phylogenetic community structure for each family using the net relatedness index (NRI) and nearest taxon index (NTI), calculated using publicly available data from the Barcode of Life Data Systems (BOLD); compares patterns across families with different traits (habitat, diet) using standard statistical analysis (ANOVA) as well as phylogenetic generalized least squares (PGLS) using a family‐level beetle phylogeny obtained from the literature; and compares community structure in Churchill with a region in southern Canada (Guelph, Ontario). These analyses were also repeated at a genus level. The dominant pattern detected in our study was that aquatic families were much better represented in Churchill compared to terrestrial families, when compared against richness sampled from across Canada and Alaska. Individually, most families showed significant phylogenetic clustering in Churchill, likely due to the strong environmental filtering present in Arctic environments. There was no significant difference in phylogenetic structure between Churchill and Guelph but with a trend toward stronger clustering in the North. Fungivores were significantly more overdispersed than other feeding modes, predators were significantly more clustered, and aquatic families showed significantly stronger clustering compared to terrestrial. This study contributes to our understanding of the traits and processes structuring insect biodiversity and macroecological trends in the sub‐Arctic.

## Graphical abstract

This study determines the phylogenetic community structure of beetles in Churchill, MB, comparing patterns across families with different traits. Churchill exhibited increased clustering, and aquatic and predaceous families were significantly more clustered than terrestrial.

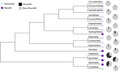

## 1 INTRODUCTION

The Arctic is a land of change (Pielou, 1995). Glaciation changed, or largely eliminated, the communities inhabiting sub‐Arctic areas such as Churchill, Manitoba, and Canada (Pielou, 1995). This region was very recently deglaciated (approximately 8K years ago), and the species composition of this area was formed by postglaciation colonization, with species primarily coming from the south and from the Beringian glacial refuge (Brandson, 2011; Pielou, 1995; Woodcock et al., 2013). While biodiversity in general tends to decrease with latitude, Arctic environments provide a diverse range of habitats and niches for life (Danks, 1992; Woodcock et al., 2013). As the climate shifts, these communities and habitats are experiencing rapid changes; this may be due to increasing temperature, melting sea ice, increased greenery, changing nutrient levels, or invading species (Walseng et al., 2018). Important questions remain about Arctic biodiversity, such as what species and traits make up Arctic communities, where did they colonize from, what patterns exist in their community structure, and how will these patterns shift in the future? With ongoing climate change, it is important to understand the traits of Arctic and sub‐Arctic species, as well as to predict how their geographic ranges and community structure may shift in the future.

Investigating evolutionary community structure can help us understand the relationships among species in Arctic communities and their distribution patterns. Phylogenetic community structure metrics are used to quantify the relatedness among cohabiting species against patterns in a broader source community (Boyle & Adamowicz, 2015; Emerson et al., 2011; Kraft et al., 2007; Mayfield & Levine, 2010; Smith et al., 2014; Webb, 2000; Webb et al., 2002). Are the species found in a local community more closely related than those in a broader community? What does this tell us about the mechanisms underlying their relationships and distributions?

In order to reconstruct and understand the phylogenetic relationships among species, it is beneficial to analyze DNA sequence data, which is a rich source of data for inferring relationships (Hillis et al., 1996). DNA barcodes are standardized DNA sequences that are used for specimen identification and species discovery (Hebert et al., 2003; Hebert & Gregory, 2005; Hubert & Hanner, 2015), and which also harbor phylogenetic signal to resolve relationships among closely related species (Boyle & Adamowicz, 2015; Smith et al., 2014; Wilson, 2010, 2011). The barcode most commonly used for animals is an approximately 658 base pair region of cytochrome c oxidase subunit I (COI), a mitochondrial gene (Adamowicz, 2015; Hebert et al., 2003). DNA barcoding allows for data to be readily available to other scientists through data banks like the Barcode of Life Data Systems (BOLD), which contains a large collection of geo‐referenced specimens from locations around the world (Ratnasingham & Hebert, 2007). This study leverages publicly available, geo‐referenced sequence data for beetles from BOLD, combined with a published multi‐gene backbone phylogeny (Zhang et al., 2018), to combine the merits of both approaches for community phylogenetics (Boyle & Adamowicz, 2015; Smith et al., 2014).

**FIGURE 1:**
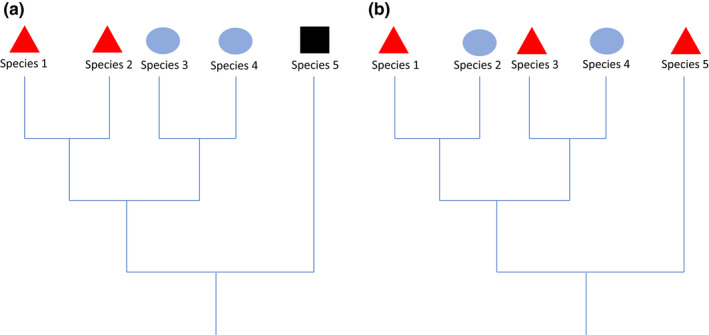
Phylogenetic trees demonstrating phylogenetic community structure patterns. Each habitat or geographic region is shown by a different colour and shape. (a) Pattern a shows a clustering pattern, where closely related species share the same region. (b) Pattern b shows an overdispersed pattern, where closely related species inhabit different regions or environments

Various patterns can occur in phylogenetic community structure, including patterns of clustering, overdispersal, or random (Webb, 2000; Webb et al., 2002). A clustered pattern occurs when closely related species are found together more often than expected by chance, often caused by environmental filtering (Figure 1a) (Emerson et al., 2011; Kraft et al., 2007; Smith et al., 2014; Weiher et al., 2011). In this case, cohabiting species typically share the traits needed to survive in a given environment and are therefore found in the same region, while more distantly related species that lack these traits are excluded. Overdispersion occurs when closely related species cohabit in the same local community less than is expected (Figure 1b) (Emerson et al., 2011; Kraft et al., 2007; Mayfield & Levine, 2010; Weiher et al., 2011). This is often interpreted as evidence for competitive exclusion, whereby closely related species compete for the same resource, and this results in one species being forced out of the environment or into a different niche (Emerson et al., 2011; Kraft et al., 2007; Weiher et al., 2011). However, it is difficult to draw conclusions about mechanisms and the causes of these patterns based on the phylogenetic patterns alone. Mayfield and Levine (2010) suggest that competitive exclusion can also cause clustering. If competitive ability is phylogenetically clustered and is more important for surviving in the environment than niche differences, we can expect competitive exclusion to cause clustering rather than overdispersion (Mayfield & Levine, 2010). In order to draw conclusions about mechanisms, it may be beneficial to examine traits rather than community phylogenetic patterns alone.

There are various environmental and biotic factors that may influence the phylogenetic structure of communities, and these may change with latitude. Factors such as the strength of competition and environmental filtering change across latitude. In northern environments, the climate and environmental factors can be important for determining species assemblages (Ernst & Buddle, 2015). Dispersal ability of various taxonomic groups, geographic barriers, and stochasticity are also expected to play a role in the recolonization of formerly glaciated northern regions.

The biological traits of the species within a community, such as diet or lifestyle, can also affect the phylogenetic structure (Mayfield & Levine, 2010). For example, Poulin et al. (2011) found that closely related parasitic species are found together in local communities more than expected, likely due to closely related species having similar hosts. If these hosts are clustered geographically, we can expect the same of the parasites (Eagalle & Smith, 2017; Poulin et al., 2011). Similarly, Vamosi and Vamosi (2007) discussed the effects of an aquatic lifestyle on community structure, with dytiscid beetle communities in the lakes of Alberta showing phylogenetic clustering. This may have been caused by a decrease in the importance of competition and an increase in environmental filtering in aquatic systems relative to terrestrial (Vamosi & Vamosi, 2007). In order to survive in aquatic environments, species need to have a certain set of physiological tolerances, and environmental factors such as salinity and pH influence the diversity (Heino et al., 2016) and composition of species found in the environment (Vamosi & Vamosi, 2007). However, different processes interact to determine species survival and co‐existence, and it may be difficult to pinpoint one cause or mechanism (Peres‐Neto et al., 2012). Across these varied examples, the lifestyles and characteristics of the species influence the community structure.

While prior studies have investigated clustering patterns and community structure within specific taxa and locations, few have compared these patterns across taxa or investigated how community structure is related to traits (Kraft et al., 2007; Poulin et al., 2011; Vamosi & Vamosi, 2007; Weiher et al., 2011). In this study, we investigate the community composition of a sub‐Arctic region and seek to determine if phylogenetically related species are more likely to have colonized this area or if the community composition is random or overdispersed in relation to phylogeny. We investigate the patterns that occur in phylogenetic community structure at a species level across taxa and traits and investigate the phylogenetic relatedness of species inhabiting the sub‐Arctic site of Churchill, Manitoba using northern North America as the regional species pool. We also seek to determine if biological traits (habitat, diet) influence the phylogenetic community patterns. This study allows us to investigate what traits are relatively more prevalent in Arctic communities and whether families with specific traits tend to exhibit phylogenetic clustering. By understanding the current traits and community structure, and how these relate to environmental factors, we can better prepare for the changes likely to occur in the future. We hypothesize that environmental filtering will impact community structure of sub‐Arctic communities due to the harsh environmental conditions present at higher latitudes. Specifically, we predict that the species in Churchill will present a significantly clustered pattern when compared against the broader North America species phylogeny. When comparing other regions within North America, we expect the regions found at higher latitudes to show a more significant clustered pattern. Second, we hypothesize that the traits and characteristics of the species will influence the community structure. We predict that taxonomic groups with traits that expose them to more environmental filtering, such as being aquatic, or relying on a host species, such as being a parasite or parasitoid, will have a more clustered pattern than their terrestrial and free‐feeding counterparts.

## 2 METHODS

### 2.1 Data and taxa

The focal organisms for this study are sub‐Arctic Coleoptera. Beetles are understudied in previous community structure research yet are hyper‐diverse, with species occupying a variety of niches and habitats and exhibiting substantial variability in traits (Marshall, 2006; Woodcock et al., 2013). There are also 466,260 public records available on the BOLD database as of July 7th, 2021. Particularly, we will be focusing on the Churchill region as there has been a concentrated effort to barcode fauna in northern communities, particularly Churchill (Woodcock et al., 2013; Zhou et al., 2009, 2010). In the BOLD database (Ratnasingham & Hebert, 2007), there are 315 recorded species of Coleoptera in Churchill as of July 7th, 2021.

Using BOLD’s application programming interface (API), all data for this study were pulled from the BOLD database [June 19th, 2019] directly into the R environment. The code for this study is available at github.com/S‐Majoros/Phylogenetic_Community_Structure_Code.r. All coding was done in R version 3.5.0 (R Core Team, 2019). Data for both Canada and Alaska were used as the regional species pool and compared to the data from Churchill, which will be defined as the local community for this study. BINs (Barcode Index Number; Ratnasingham & Hebert, 2013) were used to represent species. BINs are OTUs (operational taxonomic units) that are clusters of barcode sequences similar to species (Ratnasingham & Hebert, 2013). We chose to use BINs to represent species in this study, because, for beetles, BINs frequently correspond to morphologically defined species boundaries (Pentinsaari et al., 2014, 2017). While BINs do not always perfectly match with recognized species boundaries, Pentinsaari et al. (2014), Pentinsaari et al. (2017) found in Coleoptera that BINs matched with species 90%–92% of the time. We propose that using BINs is a valuable approach for insect biogeographic studies due to the widespread presence of cryptic (or nearly cryptic) evolutionary lineages in insects (Smith et al., 2006). Using BINs is, at this time, likely to result in a more complete account of the biodiversity present and readily enables comparison of biodiversity between geographic regions.

### 2.2 Filtering data and defining Churchill

**FIGURE 2:**
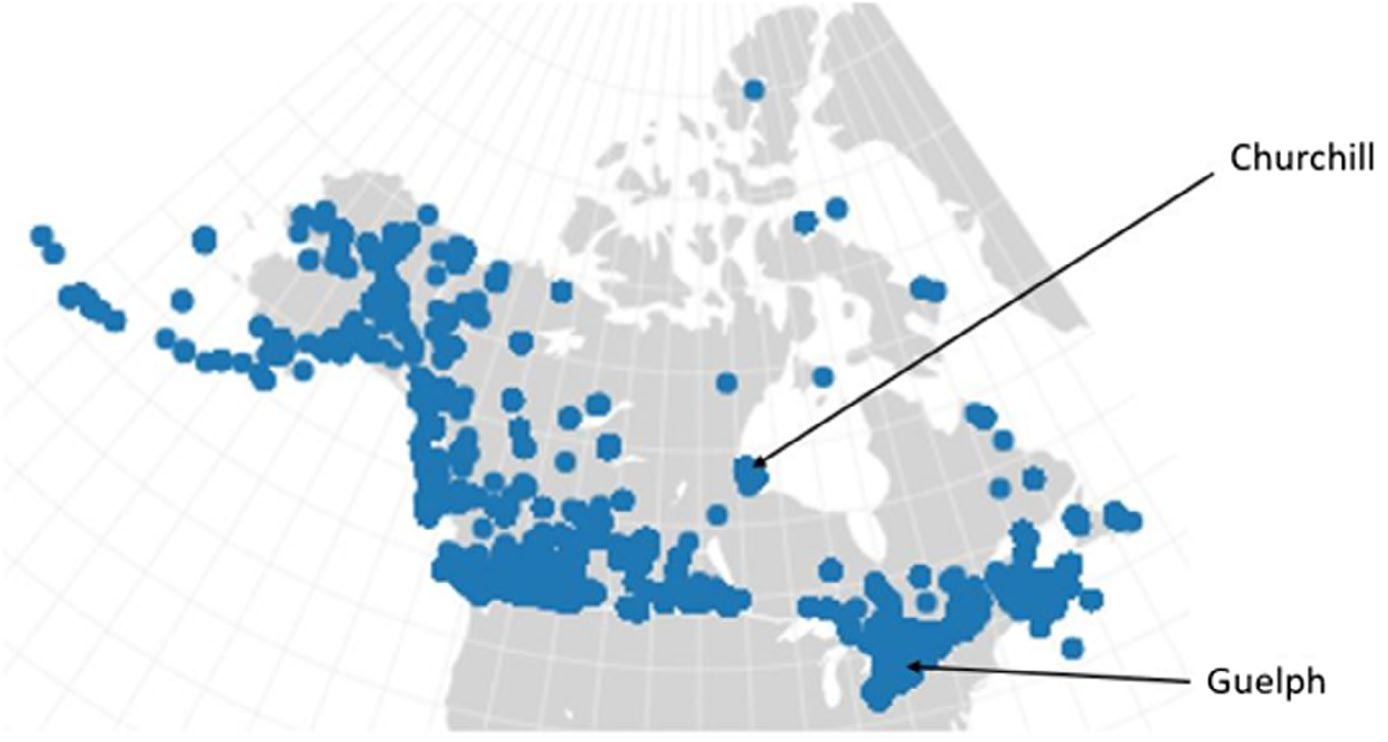
Map showing the location of Churchill and Guelph and the sampling sites within Canada and Alaska for beetle data available from BOLD

Once the sequences and metadata had been pulled from BOLD, the data were filtered. Families and genera were included in the analysis if they had three or more BINs present in Churchill. DNA sequences without a BIN assignment or GPS coordinates were removed. Sequences were also removed if they were not from the COI‐5P marker, if they had internal missing data (“N” nucleotides) or gap content greater than 1% of the sequence length, or were less than 500 base pairs. COI is commonly used for DNA barcoding animals and provides useful phylogenetic signal at low taxonomic levels but has some limitations when used to construct deep phylogenies (Boyle & Adamowicz, 2015; Smith et al., 2014; Wilson, 2010, 2011). This limited phylogenetic signal can be helped by using a constraint tree when constructing phylogenies (Boyle & Adamowicz, 2015; Smith et al., 2014; Wilson, 2011). Despite some limitations, COI can be readily sequenced from a large number of taxa and provides high sequence quality compared to other gene regions (Wilson, 2010). Barcode‐based trees have also shown similar results when used for community phylogenetics compared to other trees (Boyle & Adamowicz, 2015; Erpenbeck et al., 2007; Smith et al., 2014). Because of these findings, COI was suitable to use for this study. Additionally, this marker had the advantage of large‐scale taxonomic and geographic coverage for North American beetles (Figure 2).

The sequence datasets were reduced to one sequence per BIN for phylogenetic analysis. The sequences were first aligned within each BIN in order to choose a centroid, which is a representative sequence for each BIN, defined as the sequence with the minimum average distance to all others in its BIN (as in Orton et al., 2019). Alignments were performed using the muscle algorithm implemented in the muscle package version 3.30 (Edgar, 2004) with the following parameters: maxiters equaled 3, diags equaled true, and gapopen equaled −3000. These parameters were chosen in order to limit the number of iterations for optimization to allow for an alignment to be quickly generated, as sequences within BINs are similar. Then, the selected centroids (one per BIN) were aligned within each family. A preliminary alignment was performed for each family with the above parameters in order to trim the sequences to 658 base pairs and to screen for outliers. The sequences for each family were then aligned using a reference sequence. A reference BIN that met the following criteria was selected from the public data on BOLD: it was from the order Coleoptera, it contained at least 10 CO1‐5P sequences, it had at least one specimen photograph that matched the higher taxonomy, and it did not have taxonomic conflicts at family level or above. The reference sequence was chosen from this BIN and had to be 658 base pairs long, have 2 trace file chromatograms, and no missing information or stop codons. The reference sequence used for this study had the record id AEDNA549‐12 and was from the species *Colymbetes dolabratus*. The final alignment was performed using the same settings as the previous alignments, but with the default maxiters parameter (maxiter = 8 in R implementation using muscle package) (Edgar, 2004). The gap opening penalty is based on analyses performed by Orton et al. (2019) on taxonomic groups that contained gap regions (amino acid insertions or deletions in the COI barcode region). This gap opening penalty provided biologically realistic alignments that preserved amino acid alignment homology across taxonomic groups (Orton et al., 2019); sequences analyzed for this study were also translated in MEGA version 10.2.0 (Kumar et al., 2018) and verified to be free from stop codons. The centroid, alignment, and filtering code were adapted from publicly available code by May et al. (2020) and Orton et al. (2019).

After the data were filtered, a Churchill subset was defined using coordinates: a latitude between 58.6 and 58.7 degrees and a longitude between −94.2 and −93.8 degrees. These coordinates were found using Google Earth (Google, 2018) and based on a map provided in Boyle (2012) that showed the accessible areas in the vicinity of Churchill, MB, included in prior DNA barcoding research. This map is compatible with maps in other Churchill‐related DNA barcoding literature (Woodcock et al., 2013; Zhou et al., 2009, 2010).

### 2.3 Community phylogenetic metrics

**FIGURE 3:**
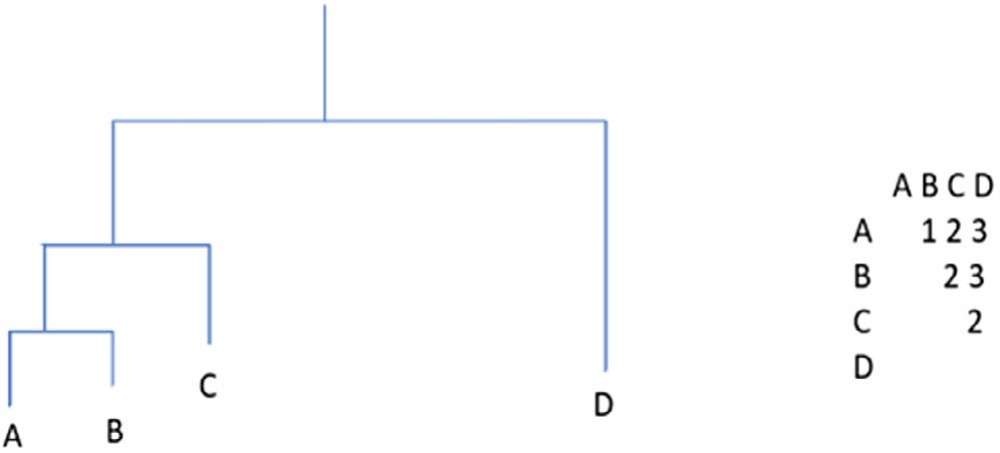
Example phylogenetic tree with a chart showing nodal distances among members of the community. NRI uses all the distances to find the mean pairwise distance ((1 + 2 + 3 + 2 + 3 + 2)/6 = 2.16). NTI uses only the distances between nearest neighbors (nearest neighbor pairs: A&B, B&A, B&C, C&D; (1 + 1 + 2 + 2)/4 = 1.5)

In order to test for phylogenetic clustering and overdispersion, we calculated net relatedness index (NRI) and nearest taxon index (NTI); the calculation of these metrics requires a phylogeny as one of the inputs. First, we generated a maximum likelihood tree for each Coleoptera family using COI one sequence per BIN for all BINs present in Canada and Alaska. The family level was chosen for analysis because members of beetle families often share important traits, such as feeding mode (Hunt et al., 2007). Before reconstructing the phylogenies, we first estimated the best‐fit model of nucleotide evolution for each family using the R package phangorn version 2.4.0 (Schliep, 2011). The model with the lowest Bayesian Information Criterion (BIC) score was chosen, and the proportion of invariant sites was determined based on the fitted model. BIC evaluates models based on posterior probability and maximum likelihood (Konishi & Kitagawa, 2008). The number of intervals of discrete gamma distribution (the k value) was set to 4. A neighbor‐joining tree (Saitou & Nei, 1987), generated using the function NJ from phangorn version 2.4.0 (Schliep, 2011), was used as the guide tree. Maximum likelihood trees based on COI were generated using the function optim.pml from phangorn version 2.4.0 (Schliep, 2011), and optNni, optGamma, and optInv were set to true. A bootstrapping analysis performed with 1000 replicates was then used to find the nodal support for each tree. For each family, an outgroup was chosen from another Coleoptera suborder and used to root each tree. Trees showing the nodal support values are included in Appendix S1. The most likely tree based on these replicates for each family was used in the NRI and NTI analysis. NRI and NTI calculate the pairwise distance between two species and use this to estimate the community relatedness (Webb, 2000). NRI averages the evolutionary distances between all pairs of tips in the community, while NTI takes only the distances between nearest neighbors (Figure 3) (Webb, 2000). When the NRI/NTI value (standardized measure) is above 0, this indicates phylogenetic clustering of the species within the community, while negative values indicate overdispersion (Webb, 2000). The two tests detect patterns at different levels within the phylogeny; therefore, in order to test for general patterns, both tests should be performed (Kraft et al., 2007). The NRI and NTI may differ in their estimates of significance, or, in some cases, even their predicted trend. If NRI suggests clustering, this is due to clustering occurring deeper within the phylogeny (Webb, 2000). For NTI, the clustering is occurring within the clades and at the tips of the phylogeny (Webb, 2000). For this study, it is beneficial to use both in order to detect clustering patterns at all levels. These calculations were performed using the R package picante version 1.7 (Kembel et al., 2010) and the null model “taxa.labels,” which indicates that random draws of the same species richness as the Churchill community were made from each family phylogeny; and NRI and NTI are re‐calculated with each randomization. The analysis was repeated 1000 times. The observed NRI and NTI values were then compared against the null distribution to obtain a *p*‐value. These tests determined whether species inhabiting the Churchill region are more significantly phylogenetically clustered or overdispersed than expected by chance, when compared against the phylogeny of DNA barcoded beetles of northern North America. A Holm–Bonferroni correction was also done for the *p*‐values in order to account for the test being performed 16 times. The NRI/NTI analysis was performed again using a maximum clade credibility consensus tree based on the bootstrapping replicates. A maximum clade credibility tree is chosen by summing the maximum likelihood values of each clade and selecting the tree with the highest overall score. This method is commonly used and creates highly resolved consensus trees (Beast2, n.d.; O'Reilly & Donoghue, 2018). This was done in order to see whether use of a consensus tree affects the results, which were found to be similar (Appendix S2).

### 2.4 Trait analysis

For the trait analyses, we investigated whether families with different traits have different phylogenetic community structure, by comparing the NRI/NTI values across families exhibiting different trait categories using an ANOVA. First, we created a character matrix for each family. Characters/traits were found for each family based on the literature (references available in Appendix S3). The traits that describe the majority of members of a given family were used; this included habitat (terrestrial or aquatic) and feeding mode (predaceous, phytophagous, or fungivorous). Where adult and larval diet differed, both were included as separate traits. Habitat remained relatively consistent across larvae and adult stages. We defined terrestrial as taxa that live primarily in land habitats and aquatic as taxa that live primarily in water bodies and habitats. Predaceous taxa were defined as those who prey on other insects or animals, phytophagous taxa as those who feed primarily on plant material, and fungivores as those who feed primarily on fungi. We then used a one‐way ANOVA to compare the average phylogenetic structure (NRI or NTI metric) of families across trait categories, treating each family as an independent unit (as supported by the results of Pyle, 2018). We conducted a second analysis considering phylogenetic relationships among families. We created a family‐level phylogenetic tree, that is, treating each family as one tip, using the phylogenetic hypothesis provided in Zhang et al. (2018) based upon 95 protein‐coding genes. Five species from the order Neuroptera were chosen as outgroups. Based upon their topology, the tree was constructed manually using Mesquite (Maddison & Maddison, 2019) and loaded into R. We assigned branch lengths of 1, before fitting a phylogenetic generalized least squares (PGLS) model using picante version 1.7 (Kembel et al., 2010). This allowed us to determine whether families with particular traits have different clustering patterns while taking into account the relationships among families. The PGLS analysis used Brownian motion as the model of trait evolution, and the log‐likelihood was maximized for the method. A chi‐square test was also performed to determine whether the proportional representation of BINS in Churchill varied with traits.

### 2.5 Community phylogenetic metrics for a temperate region

In order to compare the phylogenetic community structure patterns in Churchill to a temperate location, the analysis above was repeated for the Guelph region. Guelph was selected due to its temperate climate and the abundance of data available on the BOLD database (Ratnasingham & Hebert, 2007). A Guelph subset was defined using coordinates: a latitude between 43.4 and 43.6 degrees and a longitude between −80.3 and −80.1 degrees. These coordinates were found using Google Earth (Google, 2018). In order to determine if the community structure of the Churchill and Guelph subsets were significantly different, a paired *t*‐test was performed to compare mean NTI and NRI values for beetle families between these sites. The trait analysis was also repeated for the Guelph region. Due to the increased number of families found in this region, several more categories were added to feeding mode. Saprophagous taxa were defined as those that feed primarily on decaying organic matter, and omnivores as those that feed relatively equally on both plant and animal matter.

### 2.6 Analysis at a genus level

The analyses described above were repeated at the genus level for both the Churchill and Guelph regions. Genera needed to have three or more BINs present in Churchill to be included. The data were filtered using the same criteria as the data at the family level, NRI/NTI was calculated for each genus, and traits were assigned at the genus level, instead of the family level as described above (references in Appendix S3). Traits are able to be assigned more accurately at the genus level, that is, with less variability among species within genera than among species within families. Some additional trees were needed in order to find the relationships among beetle genera. In combination with Zhang et al. (2018), Michat et al. (2017) was used for Dytiscidae, Nie et al. (2018) for Chrysomelidae, and Gusarov (2018) for Staphylinidae.

### 2.7 Sensitivity analysis: constraint tree

An important part of phylogenetic analysis is the phylogenetic tree, and NRI/NTI and PGLS are all affected by the phylogenetic tree used. As discussed earlier, there are some issues with generating phylogenetic trees using COI data alone, although reasonable phylogenetic signal is expected among close relatives (Wilson, 2010, 2011). Park et al. (2018) suggested that inferred phylogenies often underestimate phylogenetic diversity, and errors in the phylogenetic reconstruction are common when environmental filtering is present, which we expect here. It is important to choose good constraint trees and outgroups. In order to account for the issues associated with the use of COI in the generation of phylogenetic trees, a sensitivity analysis was performed that used a constraint tree in addition to COI to generate Maximum Likelihood trees for individual families. The use of a constraint tree plus COI data is gaining support for constructing species‐level phylogenies in diverse insect groups, including in caddisflies (Boyle & Adamowicz, 2015) and ants (Smith et al., 2014). However, due to limitations in the literature, a full constraint tree cannot be constructed for each family and genus. One family and one genus were chosen for the constraint analysis: Dytiscidae and *Agonum* (from the family Carabidae). Species‐level constraint trees were built using trees from the literature: Michat et al. (2017) and Zimmerman (1981) for Dytiscidae and Liebherr and Schmidt (2004) for *Agonum*. For the Dytiscidae tree, 5 sequences from the genus *Cicindela* from the closely related family Carabidae were chosen as outgroups. For *Agonum*, 5 species from the closely related genus *Amara* were chosen as outgroups. Constraint trees were constructed manually using Mesquite (Maddison & Maddison, 2019), and the alignments were generated in R as described above. The maximum likelihood trees were generated using RAxML (Stamatakis, 2014) using the COI sequence data with a binary (i.e., bifurcating) constraint tree. The most common species‐level identification for each BIN was used to assign species‐level taxonomy to each BIN. These trees were then imported into R in order to complete the NRI/NTI analysis.

### 2.8 Sensitivity analysis: size of regional species pool and taxon richness of source pool

Kraft et al. (2007) state that the power for the NRI and NTI analysis is highest when local species richness is 30%–60% of regional species richness. For the Coleoptera of Churchill, all families are below this range except for Dytiscidae, Gyrinidae, and Haliplidae. To determine the effects of this, a sensitivity analysis was performed. The regional BIN pool was restricted to the Canadian provinces and territories of Manitoba, Nunavut, Northwest Territories, Saskatchewan, and Ontario. This restriction also helps combat some patterns that may be based on biogeography. For example, the Rocky Mountain Range may act as a barrier to dispersal, and this could create a clustering pattern on its own. By restricting the regional pool, we can largely control this effect. The same families and phylogenetic tree were used in this analysis. A paired *t*‐test was then performed to compare the results of the NRI/NTI using the restricted BIN pool to the original analysis.

## 3 RESULTS

### 3.1 Phylogenetic clustering metrics

**FIGURE 4:**
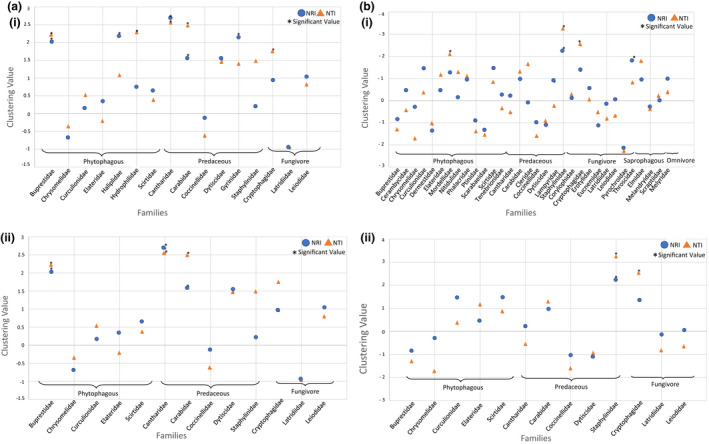
(a) (i) Phylogenetic community metrics for Coleoptera families in Churchill, MB. (ii) Shows only the families also present in Guelph for ease of comparison. A positive value indicates a clustered pattern, and a negative value marks an overdispersed pattern. Families exhibiting significant (*p*‐value < .05) clustering are marked by an asterisk. The majority of families tend toward a clustering pattern. (b) Graph showing the clustering values for Coleoptera families in Guelph, ON. The phylogenetic community structure is generally random, without a clear trend toward overdispersion or clustering. Families are more overdispersed in this region than Churchill. (ii) Shows only the families also present in Churchill for ease of comparison. For all the graphs, families are organized by adult diet

Sixteen families of Coleoptera were analyzed for the study, following the data filtering steps described above, of which seven showed significant phylogenetic clustering (full results in Table 1a; Figure 4a). After applying the Holm–Bonferroni correction, Cantharidae (original *p* = .001, corrected *p* = .016) was the only family still showing statistical significance. This suggests that, while the results for Cantharidae are very significant, there could be some false positives in the other families, which did not meet this threshold. This analysis was repeated using a maximum clade credibility tree, and the results for some families did differ. However, there was no significant difference between the clustering values generated with the consensus tree and without (*t*‐statistic = 1.78, *p* = .09; full results available in Appendix S2). A genus‐level analysis was also completed for Churchill. Following the same data filtering steps as the family level, 11 genera were included in the analysis, five of which showed a nonsignificant trend toward clustering (full results in Table 1b).

**TABLE 1:** Community phylogenetic structure and other metrics for (a) each of the Coleoptera families from Churchill, (b) each of the genera from Churchill, (c) each of the Coleoptera families from Guelph, and (d) each of the genera from Guelph

(a)												
Family	Number of BINs in Canada and Alaska	Number of BINs in Churchill	% of Total found in Churchill	Number of Sequences in Canada and Alaska	Number of Sequences in Churchill	Habitat	Adult feeding mode	Larval feeding mode	Clustering value NRI	*p*‐Value NRI	Clustering value NTI	*p*‐Value NTI
Buprestidae	87	3	4	470	3	Terrestrial	Phyto	Phyto	**2.04**	.**038**	**2.2**	.**05**
Cantharidae	101	6	6	5043	23	Terrestrial	Pred	Pred	***2.68***	. ***001***	**2.54**	.**007**
Carabidae	418	20	5	3642	90	Terrestrial	Pred	Pred	**1.57**	.**05**	**2.47**	.**013**
Chrysomelidae	264	5	2	3805	71	Terrestrial	Phyto	Phyto	−0.69	.75	−0.38	.63
Coccinellidae	108	4	5	2481	9	Terrestrial	Pred	Pred	−0.13	.57	−0.65	.75
Cryptophagidae	68	3	5	428	5	Terrestrial	Fung	Fung	0.95	.15	**1.73**	.**05**
Curculionidae	364	8	2	7453	11	Terrestrial	Phyto	Phyto	0.16	.45	0.51	.27
Dytiscidae	120	36	43	1531	140	Aquatic	Pred	Pred	1.52	.07	1.45	.07
Elateridae	251	5	2	3035	20	Terrestrial	Phyto	Phyto	0.35	.38	−0.23	.55
Gyrinidae	25	7	39	215	22	Aquatic	Pred	Pred	**2.15**	.**03**	1.39	.09
Haliplidae	15	6	67	75	6	Aquatic	Phyto	Phyto	**2.19**	.**02**	1.07	.16
Hydrophilidae	62	6	11	265	13	Aquatic	Phyto	Pred	0.74	.22	**2.26**	.**02**
Latridiidae	83	3	4	4216	11	Terrestrial	Fung	Fung	−0.96	.83	−0.98	.85
Leiodidae	129	5	4	593	19	Terrestrial	Fung	Fung	1.03	.15	0.79	.21
Scirtidae	46	3	7	2881	9	Aquatic	Phyto	Phyto	0.65	.22	0.36	.28
Staphylinidae	972	21	2	7187	35	Terrestrial	Pred	Pred	0.2	.43	1.46	.08

(b)											
Genus	Number of BINs in Canada and Alaska	Number of BINs in Churchill	% of Total found in Churchill	Number of sequences in Canada and Alaska	Number of sequences in Churchill	Habitat	Feeding mode	Clustering value NRI	*p*‐Value NRI	Clustering value NTI	*p*‐Value NTI
*Agabus*	19	9	47	353	35	Aquatic	Pred	1.66	.063	−0.13	.55
*Agonum*	28	4	14	226	6	Terrestrial	Pred	−0.14	.517	0.15	.43
*Coelambus*	11	3	27	139	16	Aquatic	Pred	1.05	.214	0.62	.3
*Contacyphon*	33	3	9	2547	9	Aquatic	Phyto	0.18	.4	−0.1	.45
*Cymindis*	7	3	42	53	5	Terrestrial	Pred	1.35	.18	1.25	.19
*Dichelotarsus*	23	5	22	887	17	Terrestrial	Pred	1.68	.058	1.08	.15
*Gyrinus*	20	7	35	172	22	Aquatic	Pred	0.78	.21	0.29	.38
*Haliplus*	12	6	50	70	6	Aquatic	Phyto	1.14	.096	−0.35	.61
*Hydroporous*	21	8	38	220	25	Aquatic	Pred	0.16	.43	−0.14	.55
*Ilybius*	13	3	23	168	13	Aquatic	Pred	−0.91	.84	−0.45	.7
*Rhantus*	6	3	50	77	7	Aquatic	Pred	0.53	.202	0.44	.26

(c)												
Family	Number of BINs in Canada and Alaska	Number of BINs in Guelph	% of Total found in Guelph	Number of sequences in Canada and Alaska	Number of sequences in Guelph	Habitat	Adult feeding mode	Larval feeding mode	Clustering value NRI	*p*‐Value NRI	Clustering value NTI	*p*‐Value NTI
Buprestidae	87	5	6	470	7	Terrestrial	Phyto	Phyto	−0.87	.81	−1.34	.95
Cantharidae	101	10	10	5043	87	Terrestrial	Pred	Pred	0.22	.38	−0.56	.72
Carabidae	418	21	5	3642	38	Terrestrial	Pred	Pred	0.96	.16	1.27	.1
Cerambycidae	248	17	7	2136	36	Terrestrial	Phyto	Phyto	0.46	.33	−0.47	.66
Chrysomelidae	264	61	23	3805	280	Terrestrial	Phyto	Phyto	−0.31	.63	−1.76	.96
Cleridae	38	9	24	631	51	Terrestrial	Pred	Pred	−0.11	.51	1.6	.07
Coccinellidae	108	18	17	2481	231	Terrestrial	Pred	Pred	−1.01	.84	−1.64	.95
Corylophidae	22	4	18	449	59	Terrestrial	Fung	Fung	0.15	.4	0.22	.36
Cryptophagidae	68	8	12	428	14	Terrestrial	Fung	Fung	1.35	.07	**2.5**	.**01**
Curculionidae	364	36	10	7453	364	Terrestrial	Phyto	Phyto	1.44	.07	0.33	.36
Dermestidae	19	4	21	51	8	Terrestrial	Phyto	Phyto	−1.38	.94	−1.08	.1
Dytiscidae	120	4	3	1531	4	Aquatic	Pred	Pred	−1.09	.88	−0.97	.85
Elateridae	251	14	6	3035	43	Terrestrial	Phyto	Phyt	0.47	.33	1.12	.12
Elmidae	11	4	36	175	4	Aquatic	Sapro	Sapro	0.95	.14	1.75	.07
Erotylidae	18	6	33	294	64	Terrestrial	Fung	Fung	0.57	.24	0.01	.47
Eucnemidae	23	8	35	219	15	Terrestrial	Fung	Fung	−1.14	.87	−0.53	.69
Lampyridae	25	6	24	1044	27	Terrestrial	Pred	Pred	0.86	.28	−0.28	.59
Latridiidae	83	18	22	4216	340	Terrestrial	Fung	Fung	−0.12	.52	−0.86	.8
Leiodidae	129	9	7	593	42	Terrestrial	Fung	Fung	0.03	.49	−0.69	.75
Melandryidae	36	5	14	489	6	Terrestrial	Sapro	Sapro	−0.3	.64	−0.4	.64
Melyridae	41	3	7	325	5	Terrestrial	Omni	Omni	0.97	.15	0.33	.3
Mordellidae	101	26	26	834	86	Terrestrial	Phyto	Phyto	1.24	.12	**2.07**	.**02**
Nitidulidae	59	11	19	936	30	Terrestrial	Phyto	Phyto	0.16	.41	1.25	.11
Phalacridae	19	5	26	510	62	Terrestrial	Phyto	Phyto	0.93	.19	1.06	.15
Ptinidae	54	4	7	298	5	Terrestrial	Phyto	Phyto	−0.93	.81	−1.43	.94
Pyrochroidae	14	3	21	180	6	Terrestrial	Fung	Fung	−2.16	.99	−2.3	.98
Scarabaeidae	81	7	9	361	17	Terrestrial	Phyto	Sapro	−1.36	.91	−1.59	.95
Scirtidae	46	8	17	2881	173	Aquatic	Phyto	Phyto	1.47	.08	0.82	.23
Scraptiidae	37	3	8	1535	4	Terrestrial	Sapro	Sapro	0.04	.54	0.16	.43
Staphylinidae	972	89	9	7180	198	Terrestrial	Pred	Pred	**2.24**	.**01**	**3.23**	.**001**
Tenebrionidae	54	4	7	329	25	Terrestrial	Phyto	Phyto	0.25	.4	−0.37	.63
Throscidae	19	9	47	1651	492	Terrestrial	Fung	Fung	**1.83**	.**03**	0.79	.24

(d)											
Genus	Number of BINs in Canada and Alaska	Number of BINs in Guelph	% of Total found in Guelph	Number of sequences in Canada and Alaska	Number of sequences in Guelph	Habitat	Feeding mode	Clustering value NRI	*p*‐Value NRI	Clustering value NTI	*p*‐Value NTI
*Aleochara*	20	5	25	106	7	Terrestrial	Pred	−1.51	.93	−0.88	.79
*Amischa*	5	3	60	199	17	Terrestrial	Pred	−1.11	.76	−1.32	.84
*Ampedus*	45	3	7	398	4	Terrestrial	Phyto	0.64	.31	0.14	.43
*Atomaria*	35	5	14	228	9	Terrestrial	Fung	0.9	.17	0.96	.19
*Chaetocnema*	12	3	25	81	6	Terrestrial	Phyto	**1.97**	.**04**	**2.37**	.**03**
*Contacyphon*	33	7	21	2547	172	Aquatic	Phyto	1.28	.11	0.42	.35
*Corticarina*	15	3	20	535	69	Terrestrial	Fung	−0.64	.73	−0.93	.74
*Crepidodera*	8	3	38	143	8	Terrestrial	Phyto	0.42	.2	0.57	.26
*Cryptophagus*	20	3	15	125	5	Terrestrial	Fung	1.11	.14	1.6	.13
*Enoclerus*	9	4	44	370	35	Terrestrial	Pred	−0.67	.75	0.68	.29
*Epitrix*	6	3	50	44	6	Terrestrial	Phyto	−0.19	.66	0.02	.67
*Glischrochilus*	8	6	75	465	17	Terrestrial	Phyto	−0.56	.63	−0.33	.73
*Hippodamia*	9	3	33	139	12	Terrestrial	Pred	−0.17	.52	−0.36	.55
*Isorhipis*	4	3	75	54	10	Terrestrial	Phyto	−1.23	.88	−1.19	.87
*Lebia*	11	3	27	103	4	Terrestrial	Pred	−0.35	.66	−0.69	.78
*Longitarsus*	13	5	38	292	53	Terrestrial	Phyto	**1.84**	.**04**	**2.57**	.**01**
*Lordithon*	12	4	33	699	7	Terrestrial	Pred	0.57	.26	0.73	.19
*Melanophthalma*	18	7	39	1011	188	Terrestrial	Fung	0.84	.2	0.49	.3
*Mordellina*	19	8	42	188	38	Terrestrial	Phyto	−0.65	.71	1.31	.1
*Mordellistena*	50	13	26	293	31	Terrestrial	Phyto	1.49	.07	1.15	.14
*Oxypoda*	30	3	10	264	6	Terrestrial	Pred	−0.6	.73	−0.28	.57
*Philhygra*	25	4	16	125	7	Terrestrial	Pred	**1.88**	.**04**	0.72	.22
*Philonthus*	32	10	31	291	20	Terrestrial	Pred	−0.81	.8	−0.85	.2
*Phyllotreta*	14	5	36	517	44	Terrestrial	Phyto	0.04	.5	−0.58	.67
*Podabrus*	16	4	25	559	18	Terrestrial	Pred	0.19	.45	0.49	.32
*Polydrusus*	4	3	75	897	217	Terrestrial	Phyto	1.64	.13	1.31	.13
*Psylliodes*	6	3	50	164	81	Terrestrial	Phyto	−1.62	.98	−1.22	.93
*Psyllobora*	8	3	38	1001	19	Terrestrial	Fung	0.63	.44	0.48	.55
*Quedius*	32	3	9	232	6	Terrestrial	Pred	0.88	.17	1.28	.13
*Sepedophilus*	14	4	29	86	5	Terrestrial	Fung	−0.04	.53	0.1	.45
*Tachyporus*	19	6	32	421	27	Terrestrial	Pred	0.43	.34	0.93	.17
*Trixagus*	14	7	50	1413	489	Terrestrial	Fung	**2.69**	.**01**	1.23	.12

Note

Significant values (*p* < .05) are in bold. Significant values under the Holm–Bonferroni threshold are in italics. Feeding modes are represented in short form; Phyto = Phytophagous, Pred = Predaceous, Fung = Fungivore, Sapro = Saprophagous, and Omni = Omnivore.

The same analysis was completed for the Guelph subset. Thirty‐two families were analyzed, four of which showed significant phylogenetic clustering (full results in Table 1c; Figure 4b). Overall, Guelph appears to be more overdispersed than Churchill; however, the taxonomically paired NRI values (*t*‐statistic = 1.19, *p* = .26) and NTI values (*t*‐statistic = 1.64, *p* = .13) of the two subsets were not significantly different. For the Guelph genus‐level analysis, 32 genera were analyzed, four of which showed significant phylogenetic clustering (full results in Table 1d). A paired *t*‐test could not be done to compare Churchill and Guelph genera, as there were no shared genera between the two regions that met our inclusion criteria.

### 3.2 Trait analysis

**FIGURE 5:**
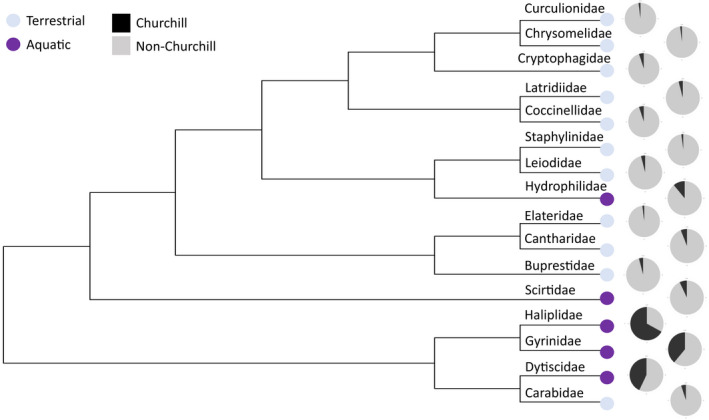
Phylogenetic tree showing the terrestrial and aquatic families present in Churchill. This tree is based on the one shown in Zhang et al. (2018). The pie graphs show the percent of the total BINs from Canada and Alaska that have been found in Churchill. Aquatic families have a larger percent of their total BINs found in Churchill than terrestrial families

**FIGURE 6:**
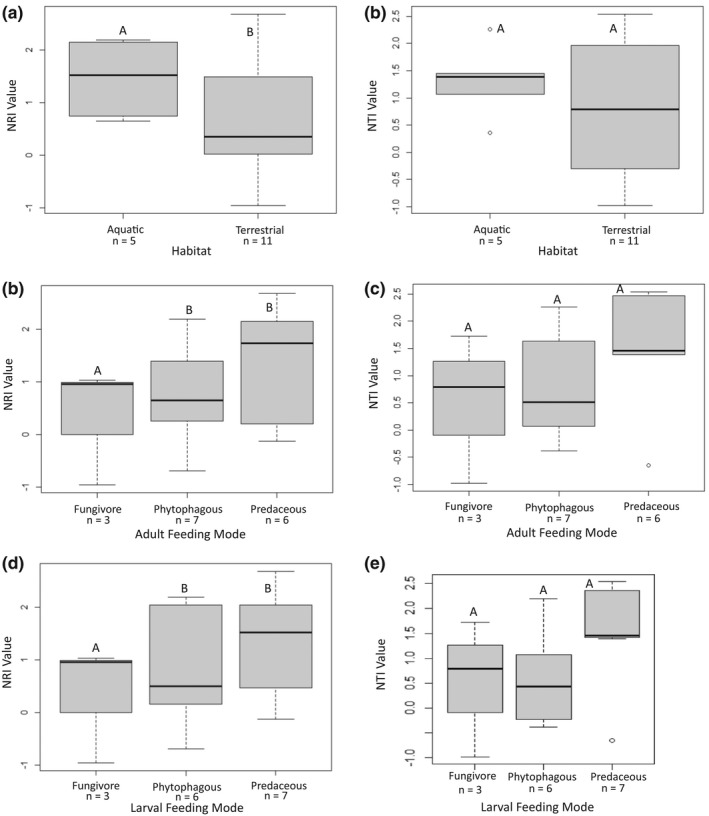
Boxplots showing the results of the PGLS for the clustering values of Coleoptera families inhabiting specific habitats using (a) NRI and (b) NTI, and the clustering values of Coleoptera families exhibiting different adult feeding modes using (c) NRI and (d) NTI, and larval feeding modes using (e) NRI and (f) NTI. The same letter above bars denotes groups that do not differ significantly, while different letters denote a significant difference; aquatic families are significantly clustered using the NRI, and fungivore families are significantly overdispersed using the NRI

Within the families studied in Churchill, only 5 were aquatic, while 11 were terrestrial. However, aquatic families have a larger percent of their total BINs found in Churchill (Figure 5; *Χ*‐squared_1_ = 76.33, *p* = 2.2 × 10^−16^). A similar result was shown for feeding mode, with the count of BINs present in Churchill, in relation to total northern North American BIN richness, differing among families with different feeding modes (*Χ*‐squared_2_ = 68.837, *p* = 1.13 × 10^−15^). At the adult life stage, seven families were phytophagous, six were predaceous, and three were fungivores; at the larval stage, six families were phytophagous, seven were predaceous, and three were fungivores. The ANOVA showed no significant relationship between the community structure metrics and the traits of the families (Table 2a), including for habitat (*F*_1.14_ = 1.79, *p* = .203), adult feeding mode (*F*_2.13_ = 1.071, *p* = .37), and larval feeding mode (*F*_2.13_ = 0.89, *p* = .43). These results were consistent with both the NRI and NTI values. The results of the PGLS differed from that of the ANOVA. Community structure was significantly related to both feeding mode and habitat (Table 2b, Figure 6). Aquatic families were significantly more clustered than terrestrial in NRI (*t* = 2.32, *p* = .04) but not NTI (*t* = 1.8, *p* = .09). Fungivore families were significantly more overdispersed than other feeding modes in NRI (*t* = 2.12, *p* = .05) but not NTI (*t* = 1.35, *p* = .2). Predators were significantly more clustered than other feeding modes in NTI (*t* = 2.43, *p* = .03) but not NRI (*t* = 0.15, *p* = .88). This result was significant only for larval feeding mode.

**TABLE 2:** The results from the (a) ANOVA, (i) for the NRI values and (ii) for the NTI values for Churchill families

	df	Sum Sq	Mean Sq	*F* value	Pr(>*F*)
(a) (i) ANOVA: NRI values					
Habitat	1	1.1	2	1.79	.203
Residuals	14	15.65	1.12		
Adult diet	2	2.5	1.25	1.071	.37
Residuals	13	15.15	1.17		
Larval diet	2	2.13	1.066	0.89	.43
Residuals	13	15.51	1.19		
(ii) ANOVA: NTI values					
Habitat	1	0.68	0.68	0.51	.49
Residuals	14	18.73	1.34		
Adult diet	2	2.1	1.05	0.79	.48
Residuals	13	17.32	1.33		
Larval diet	2	3.92	1.96	1.65	.23
Residuals	13	15.5	1.19		

	Value	Std. error	*t*‐Value	*p*‐Value
(b) (i) PGLS: NRI values				
Habitat				
Aquatic	1.45	0.63	2.32	.**04**
Terrestrial	−0.17	0.66	−0.26	.8
Adult diet				
Phytophagous	−0.42	0.57	−0.74	.47
Predaceous	0.1	0.57	0.18	.86
Fungivore	1.52	0.72	2.12	.**05**
Larval diet				
Phytophagous	−0.54	0.6	−0.9	.38
Predaceous	0.08	0.54	0.15	.88
Fungivore	1.55	0.71	2.19	.**05**
(ii) PGLS: NTI values				
Aquatic	1.39	0.77	1.8	.09
Terrestrial	−0.31	0.81	−0.39	.7
Adult diet				
Phytophagous	−0.27	0.7	−0.38	.7
Predaceous	0.36	0.7	0.51	.62
Fungivore	1.19	0.88	1.35	.2
Larval diet				
Phytophagous	0.52	0.6	0.86	.4
Predaceous	1.42	0.58	2.43	.**03**
Fungivore	0.11	0.73	0.16	.88

Note

There is no significant relationship between the phylogenetic community structure within families and the habitat or feeding mode of the families. These results differed from the (b) PGLS analysis comparing community structure within families to feeding mode and comparing community structure to habitat for both (i) NRI values and (ii) NTI values, taking into account the family‐level phylogeny of beetles. There was a significant relationship between community structure and feeding mode, as well as with habitat, though in NRI only. Aquatic families were significantly more clustered, as were predaceous families, while fungivore families were significantly more overdispersed.

In the genus‐level analysis, eight genera were aquatic, and only three were terrestrial. For feeding mode, two genera were phytophagous, and nine were predaceous. The ANOVA showed no significant relationship between the community structure metrics and the traits of the genera, including for habitat (*F*_1.9_ = 0.482, *p* = .505) and feeding mode (*F*_1.9_ = 0.001, *p* = .971). These results were consistent with both the NRI and NTI values. The PGLS analysis yielded similar results. However, there was a nonsignificant trend toward terrestrial and predaceous genera being more clustered.

The trait analyses were also performed for the Guelph region. Twenty nine families in Guelph were terrestrial, while only 3 families were aquatic. At the adult life stage, 13 families were phytophagous, seven were predaceous, eight were fungivores, three were saprophagous, and one was an omnivore, and at the larval life stage, twelve families were phytophagous, seven were predaceous, eight were fungivores, four were saprophagous, and one was omnivorous. The ANOVA showed no significant relationship between the community structure metrics and habitat (*F*_1.30_ = 0.202, *p* = .657) or feeding mode at the adult stage (*F*_4.27_ = 0.179, *p* = .947) or larval stage (*F*_4.27_ = 0.281, *p* = .888). These results were consistent across NRI and NTI and the PGLS analysis. There was a slight nonsignificant trend toward increased clustering in aquatic families.

At the genus level, 31 genera were terrestrial, and only one genus was aquatic, so no statistical test was performed for habitat. For feeding mode, 13 genera were phytophagous, twelve were predaceous, and seven were fungivores, with no difference in phylogenetic community structure found among these groups using an ANOVA (*F*_2.29_ = 1.602, *p* = .219). These results were consistent across NRI and NTI and the PGLS analysis.

### 3.3 Tree nodal support

The median nodal support value was calculated for each set of bootstrap trees. For the Churchill family level trees, the mean median nodal support value was 48.5. For the genus level, the mean median nodal support value was 55.73. For the Guelph family level trees, the mean median nodal support value was 49.25. For the genus level, the mean median nodal support value was 55.8. Median nodal support values for each individual tree can be found in Appendix S1. Typically, node support values improved toward the tips, suggesting that NTI estimates are more reliable than NRI estimates for this dataset.

### 3.4 Sensitivity analysis: constraint tree

The NRI and NTI analyses were performed again using a constraint tree for the family Dytiscidae and the genus *Agonum*. Dytiscidae with the constraint was significantly clustered, while Dytscidae without the constraint was not. *Agonum* was overdispersed in the NTI as well as NRI with the constraint tree, while without constraint *Agonum* was only overdispersed in NRI.

### 3.5 Sensitivity analysis: size of regional bin pool and taxon richness of source pool

**FIGURE 7:**
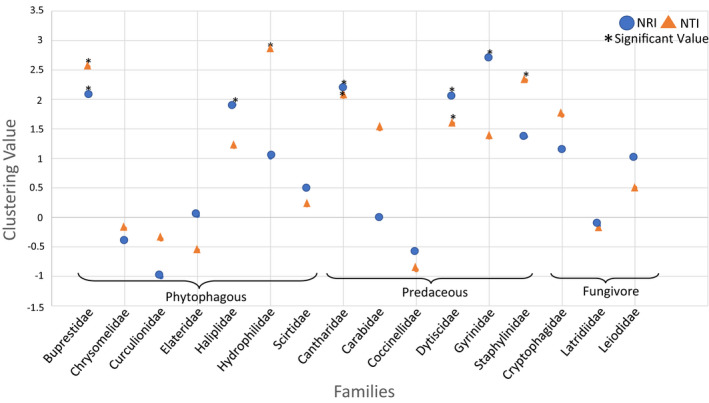
Clustering values for Coleoptera families in Churchill, MB, after the sensitivity analysis. The results did not substantially differ from the original analysis, and the main conclusions were supported

After the regional pool was reduced, Dytiscidae, Haliplidae, and Gyrinidae were still the only families where local species richness was close to 30%–60% of regional species richness (Table 3). The results for NRI and NTI did not substantially differ from the original analysis (Figure 7). This was confirmed with a paired *t*‐test comparing the NRI and NTI values between the original and restricted source phylogenies (NRI: *t*‐statistic = 0.189, *p* = .85. NTI: *t*‐statistic = 0.16, *p* = .87). Significance differed from the original analysis for some families. Staphylinidae exhibited significant evidence of clustering in NTI, and Dytiscidae exhibited significant evidence of clustering in both metrics. Carabidae lost its significance in both metrics and showed overdispersion in NRI. Cryptophagidae also lost its significance. The trends (clustering vs. phylogenetic overdispersion) remained the same for all families except Curculionidae, which became overdispersed.

**TABLE 3:** Phylogenetic community structure results for the Coleoptera families after restricting the total BIN source pool

Family	Number of BINs in Canada and Alaska	Number of BINs in Churchill	% of Total found in Churchill	Number of sequences in regional species pool	Number of sequences in Churchill	Habitat	Adult feeding mode	Larval feeding mode	Clustering value NRI	*p*‐Value NRI	Clustering value NTI	*p*‐Value NTI
Buprestidae	45	3	7	159	3	Terrestrial	Phyto	Phyto	**2.1**	.**03**	**2.55**	.**03**
Cantharidae	59	6	10	1483	23	Terrestrial	Pred	Pred	**2.19**	.**002**	**2.05**	.**01**
Carabidae	212	20	9	1702	90	Terrestrial	Pred	Pred	−0.02	.5	1.5	.07
Chrysomelidae	199	5	3	2903	71	Terrestrial	Phyto	Phyto	−0.39	.64	−0.19	.53
Coccinellidae	70	4	6	1113	9	Terrestrial	Pred	Pred	−0.56	.7	−0.89	.83
Cryptophagidae	34	3	9	203	5	Terrestrial	Fung	Fung	1.14	.11	1.73	.06
Curculionidae	204	8	4	3711	11	Terrestrial	Phyto	Phyto	−1.01	.86	−0.37	.6
Dytiscidae	51	36	71	1368	140	Aquatic	Pred	Pred	**2.08**	.**01**	**1.59**	.**05**
Elateridae	141	5	4	1092	20	Terrestrial	Phyto	Phyto	0.03	.49	−0.56	.7
Gyrinidae	11	7	64	178	22	Aquatic	Pred	Pred	**2.7**	.**02**	1.36	.08
Haliplidae	7	6	86	61	6	Aquatic	Phyto	Phyto	**1.91**	.**04**	1.2	.13
Hydrophilidae	40	6	15	191	13	Aquatic	Phyto	Pred	1.02	.15	**2.84**	.**007**
Latridiidae	52	3	6	2252	11	Terrestrial	Fung	Fung	−0.14	.5	−0.19	.55
Leiodidae	68	5	7	293	19	Terrestrial	Fung	Fung	1.01	.15	0.5	.28
Scirtidae	32	3	9	1734	9	Aquatic	Phyto	Phyto	0.49	.26	0.21	.32
Staphylinidae	485	31	6	2509	35	Terrestrial	Pred	Pred	1.37	.08	**2.32**	.**008**

Note

Significant values are bolded.

## 4 DISCUSSION

Overall, this study discovered several interesting patterns in the phylogenetic community structure of beetles. Most Churchill families and genera included in this analysis showed phylogenetic clustering. Habitat and diet were shown to impact community structure, with aquatic and predaceous families more strongly clustered than terrestrial families and those with other feeding modes. In comparison to Churchill (sub‐Arctic), Guelph (temperate) appeared more to be overdispersed, and traits had less of an effect on the phylogenetic community structure.

The main region of focus for this study was Churchill, MB, and based on our results several conclusions can be drawn. Overall, the strongest trend that we detected was higher representation of BINs from aquatic families in Churchill compared to terrestrial, indicating that beetles with aquatic habitat were more able to colonize this sub‐Arctic region. We also found significant clustering in seven of the Coleoptera families studied, and a trend toward clustering in five. This provides support for the hypothesis that, due to the harsh conditions present at high latitudes, environmental filtering would be strong in sub‐Arctic communities. The species present in the Churchill region possessed the traits needed to survive in this environment, while more distantly related species likely did not. This was not true for all families studied, as three families showed a trend toward overdispersal, though this was insignificant. All families were widely sampled across Canada, though less sampling was done in Northern Canada than Southern. The overdispersed families could potentially still have been experiencing clustering, just at a larger spatial scale, with closely related species being clustered in Canada. A trend toward clustering was also observed in the genus‐level phylogenies.

In order to compare phylogenetic community structure across latitude, the more temperate region of Guelph, ON, was included in the study. When comparing the Guelph and Churchill subsets, there was a nonsignificant trend toward Churchill being more phylogenetically clustered. It is possible that Guelph, at 43.5 degrees north, and Canada in general, is still far enough north to exhibit clustering. Guelph was less clustered than Churchill (58.7 degrees north) and if compared to more regions at even lower latitudes, it is possible there will be a more pronounced latitudinal difference. Temperate areas are under less extreme environmental pressures, which may result in stronger competition and more phylogenetically dispersed communities compared to polar regions. While our findings are consistent with the hypothesis that sub‐Arctic communities are experiencing greater environmental filtering, further investigation across a broader latitudinal gradient is warranted.

Our results were similar to those found in other studies. Ernst and Buddle (2015) found that assemblage structure was correlated with latitude and that climate was important for determining community structure in northern communities of beetles when species were placed in functional groupings. Similarly, Shibuya et al. (2011) found that in the beetle family Carabidae in Japan, the environmental conditions were more important for determining community patterns than competition, and there was actually very little interaction between the beetle species. Carabidae was significantly clustered in our study, in accordance with the findings of Shibuya et al. (2011). While Dytisicidae was not significantly clustered in this study, it still showed a trend toward clustering, similar to Vamosi and Vamosi (2007). Not all families exhibited this pattern. The importance of competition, as well as the strength of environmental filtering, likely differs between species, and this results in different community structure patterns, even under harsh environmental conditions. There were some families that exhibited a trend toward overdispersion. Ulrich and Fattorini (2013) found a similar pattern in Tenebrionidae and suggested that this could be due to colonization patterns. Differences in the past colonization patterns of the families could also influence the community structure.

Our study not only showed patterns in phylogenetic structure across regions at different latitudes, but it also showed patterns across families with different biological traits. Supporting our predictions, there was a significant relationship between phylogenetic community structure and habitat in the PGLS analyses in Churchill at the family level, though not in the ANOVA or at the genus level. Aquatic families were significantly more clustered than terrestrial. Competition is often less important in aquatic communities due to the strong influence of environmental factors (Heino et al., 2016; Vamosi & Vamosi, 2007). This pattern could also be influenced by the low BIN richness of some of the terrestrial families, as well as low plant species richness in the sub‐Arctic. Getting a true representation of terrestrial versus aquatic families was difficult due to the lack of variability in habitat across families and the limited families that inhabit the Churchill region. Only five of the 16 families studied were aquatic. However, these aquatic families had a significantly larger percent of their total northern North American species found in Churchill than terrestrial families. This suggests that it may be easier for aquatic species to colonize the Arctic than terrestrial. In order to better understand this pattern, other locations and taxonomic levels should be investigated. Based on the results of this study, habitat is likely one of the traits determining community structure in sub‐arctic environments.

Feeding mode is also likely a determining factor. There was a significant relationship between phylogenetic community structure and feeding mode. Fungivore families were significantly more overdispersed than other feeding modes. Fungivore was also the least abundant feeding mode. This aligns with the finding of Pyle (2018), who found that fungivores were not as successful in cold areas. It is possible that there are limits to the vegetation and fungi available in Arctic climates, therefore limiting the survival and diversity of fungivores and herbivores.

Significant results were also observed in predatory families, with predators more significantly clustered than other feeding modes. This pattern could possibly be due to the predator's reliance on their prey species. If the prey is clustered, so is the predator. However, out of the six predatory families studied, five were generalist predators (Marshall, 2006). The sixth family, Coccinellidae, consumes mostly aphids (Marshall, 2006). This is also the only predator family that showed a trend toward overdispersion. Therefore, the general diet of most of the families suggests that the clustering pattern observed is not dependent on their prey and that having a more general diet is beneficial for surviving in the Arctic. Another possible explanation is that these predators are able to survive in these northern habitats due to their cold tolerance and overwintering abilities. Predaceous families such as Coccinellidae and Carabidae have overwintering strategies that allow for survival in cold temperatures (Hamedi et al., 2013; Knapp & Saska, 2011). This includes occupying microhabitats that buffer the effects of the climate, lowering temperature thresholds for activity or increasing cryoprotectant concentrations (Hamedi et al., 2013; Knapp & Saska, 2011). If these traits are phylogenetically conserved, this would result in clustering. Diet has been shown to be important in other studies, such as Poulin et al. (2011), who found clustering in parasitic families due to their reliance on specific host species.

Despite the significant results observed at the family level, the relationship between phylogenetic community structure and traits was not as apparent at lower taxonomic levels. Using phylogenies of BINs within individual genera of Churchill, there was no relationship between the community structure and traits. Other traits not examined, such as overwintering strategies, may vary relatively little among species within genera, and therefore more random phylogenetic patterns may be expected within genera compared to families. There was still a trend toward increased clustering in predaceous genera, but there was also a trend toward increased clustering in terrestrial genera, the opposite of the pattern observed at the family level. There were eleven genera included in the genus‐level analysis; eight were aquatic, and only three were terrestrial. These eleven genera only included representatives of six of the Churchill families. The small sample size and lack of BIN diversity within genera of Churchill could have an effect on the results; thus, larger geographic regions and other taxa should also be investigated in future research.

The effects of traits were also compared between the two regions included in this study. It appears that habitat and diet have a greater effect in Churchill. While aquatic families still had a trend toward increased clustering in Guelph, this was not significant. There was not a strong trend observed in diet or with either of the traits at the genus level. As habitat and diet might not be the traits determining community structure in this region, a greater number of traits should be investigated in order to determine what factors influence community structure in Guelph.

Overall, there was support for our hypothesis that traits would impact phylogenetic community structure. The idea that traits are important for determining community structure is also supported by the literature; Mayfield and Levine (2010) suggest that phylogeny alone cannot determine community structure, and studies such as Vamosi and Vamosi (2007) have found traits such as body size to be related to community structure.

Our study encountered several limiting factors, but these present opportunities for future research. One was limited richness of BINs within some habitats and traits among the families present in Churchill, reflecting primarily the biological patterns as sampling has been relatively extensive for most families present (Pyle, 2018; Woodcock et al., 2013). There were more terrestrial species than aquatic and more phytophagous and predaceous families than fungivores; this makes it hard to compare accurately the observed phylogenetic clustering patterns in relation to the trait states. There were only 16 families studied here that met our inclusion criteria, and data were even more limited at the genus level. Future studies may expand on these results by conducting this analysis for other taxa, other geographic regions, and other traits. While this study did look at one temperate region, including more regions along a latitudinal gradient would allow us to understand better the effects of latitude and environmental conditions on communities. Moreover, by including more traits, we can discover what other traits are being filtered for in Arctic communities and how these traits are affecting phylogenetic community structure. Also, some families are understudied and under sampled (Brunke et al., 2019). While sampling in general is extensive for Churchill beetles (Pyle, 2018; Woodcock et al., 2013), families such as Scirtidae and Latridiidae are poorly understood and appear to be more diverse in Canada than the number of recorded species suggests (Brunke et al., 2019). By continuing to study Canada's insect communities, including intensive sampling at focal sites, we can further explore diversity, traits, and community structure based upon more complete sampling.

To summarize the observations of this study, most Churchill families and genera showed phylogenetic clustering, and most families were significantly clustered. Aquatic families were more clustered then terrestrial, and there was a larger percent of their total BINs found in Churchill compared to terrestrial families. Fungivore families were more overdispersed than other feeding modes, and predaceous families were more clustered. When compared to Churchill, Guelph families trended toward less phylogenetic clustering. Diet and habitat also appeared to have less effect on community structure in Guelph than in Churchill.

During postglacial colonization, species came from the south and from the Beringian glacial refugium (Pielou, 1995; Woodcock et al., 2013). Was this colonization random? The results of this study suggest that it was not. Closely related species, sharing similar traits, were found in sub‐Arctic communities, likely due to the environmental filtering occurring in this area. Arctic communities are particularly vulnerable to climate change and increasing temperatures (Danks, 1992; Walseng et al., 2018). If Arctic conditions change, it is possible that some of their extreme environmental pressures will decrease or shift, and the environmental filtering occurring in these environments will likely also change. By understanding the current community structure and the factors and traits influencing this, we can better predict how these communities are likely to change in the future. If temperate locations show less clustering than those in northern regions, as shown by the comparison of Guelph and Churchill in this study, we can expect communities to become less phylogenetically clustered as species move northward.

## CONFLICT OF INTEREST

The authors declare that there are no conflicts of interest.

## AUTHOR CONTRIBUTIONS

**Samantha E. Majoros:** Conceptualization (equal); Data curation (lead); Formal analysis (lead); Methodology (equal); Visualization (lead); Writing – original draft (lead); Writing – review & editing (equal). **Sarah Adamowicz:** Conceptualization (equal); Data curation (supporting); Formal analysis (supporting); Funding acquisition (lead); Methodology (equal); Writing – review & editing (equal).

## Supporting information

Appendix S1

Supplementary Material

## Data Availability

DNA Sequences and related data are publicly available on the Barcode of Life. Data Systems (http://boldsystems.org/). A csv file containing the process ids of sequences used in this analysis can be found in Supplementary material. This file contains sequences downloaded from the Barcode of Life Data Systems (BOLD) from Canada and Alaska. These sequence data sets are before filtering, as the final set of sequences differs for each analysis, as established by running the filtering steps in the R code. The character matrices and phylogenetic trees used in the analysis are available on Data Dryad (https://doi.org/10.5061/dryad.cjsxksn70).
