# Supplementary material for: Phylogenetic signal of sub‐arctic beetle communities
Source: Ecol Evol. 2022 Feb 16;12(2):e8520. doi: 10.1002/ece3.8520 (PMC8848465; doi:10.1002/ece3.8520)
Supplement: Supplementary file 1 — Appendix S1 [file ECE3-12-e8520-s002.docx]

Appendices

Appendix 1: Nodal Support Values.

The outgroups have been trimmed off for analysis purposes. Many trees had too many branches to be clearly read. Therefore, these trees are not shown but their median is still included. All trees are included as nexus files in the supplementary material.

Churchill Families:

Median nodal support value for Buprestidae: 47.7.

Median nodal support value for Cantharidae: 40.45.

Median nodal support value for Carabidae: 35.7.

Mean nodal support value for Chrysomelidae: 41.8.

Median nodal support value for Coccinellidae: 59.7.

Median nodal support value for Cryptophagidae: 35.

Median nodal support value for Curculionidae: 41.6.

Median nodal support value for Dytiscidae: 32.

Mean nodal support value for Elateridae: 39.


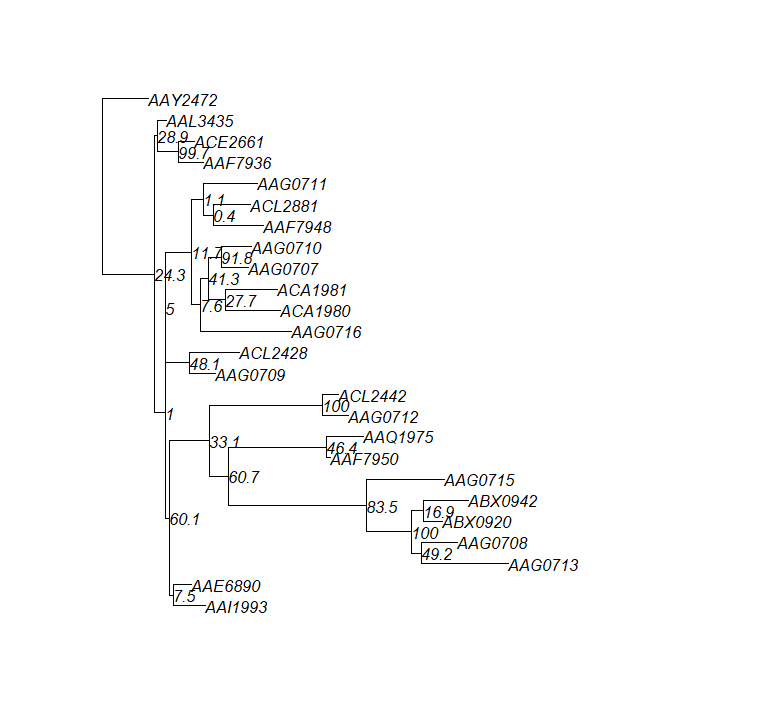


Tree showing nodal support value for Gyrinidae (median = 33.1).


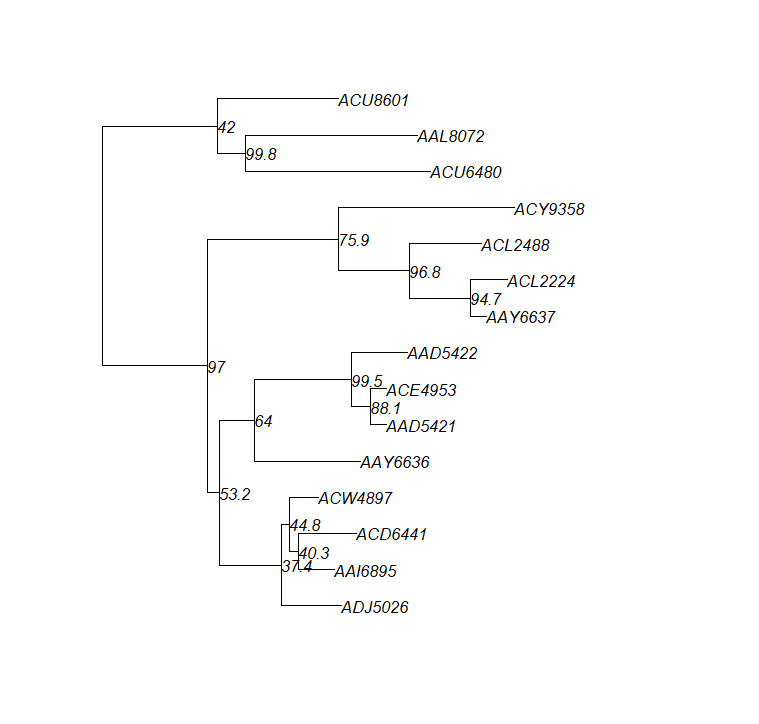


Tree showing nodal support value for Haliplidae (median = 75.9).


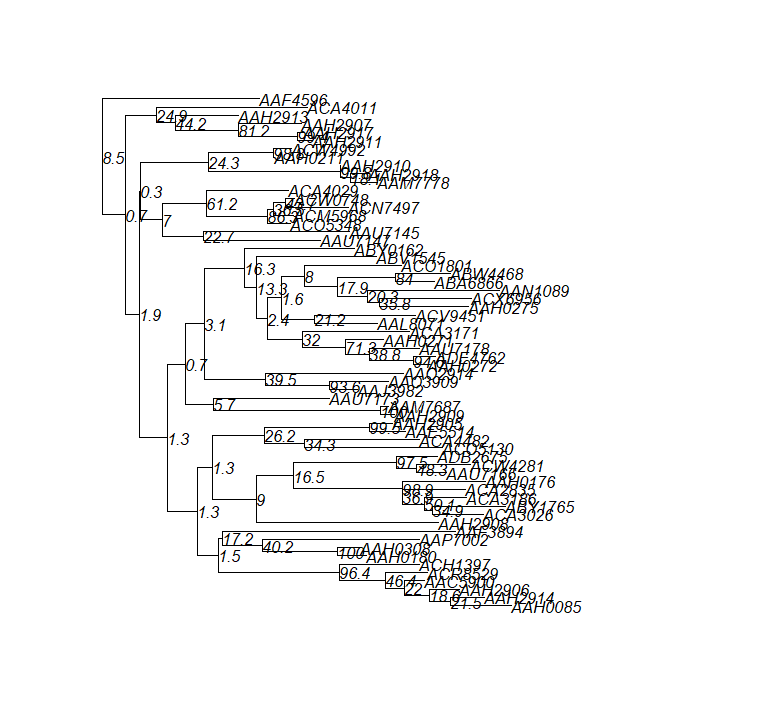


Tree showing nodal support for Hydrophilidae (median = 26.1).

Median nodal support value for Latridiidae: 53.7.

Median nodal support value for Leiodidae: 33.4.


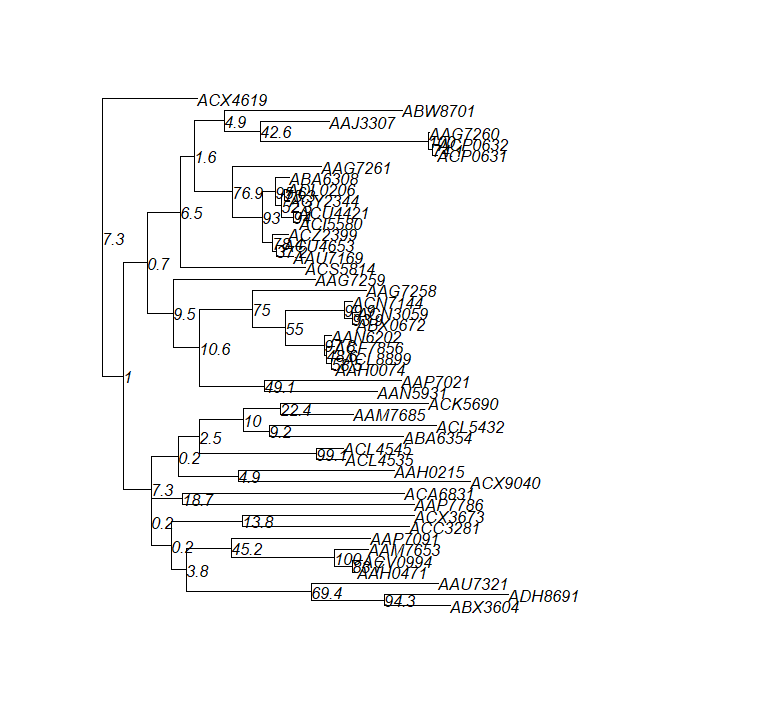


Tree showing nodal support for Scirtidae (median = 45.2).

Median nodal support value for Staphylinidae: 24.4.

Churchill Genera:


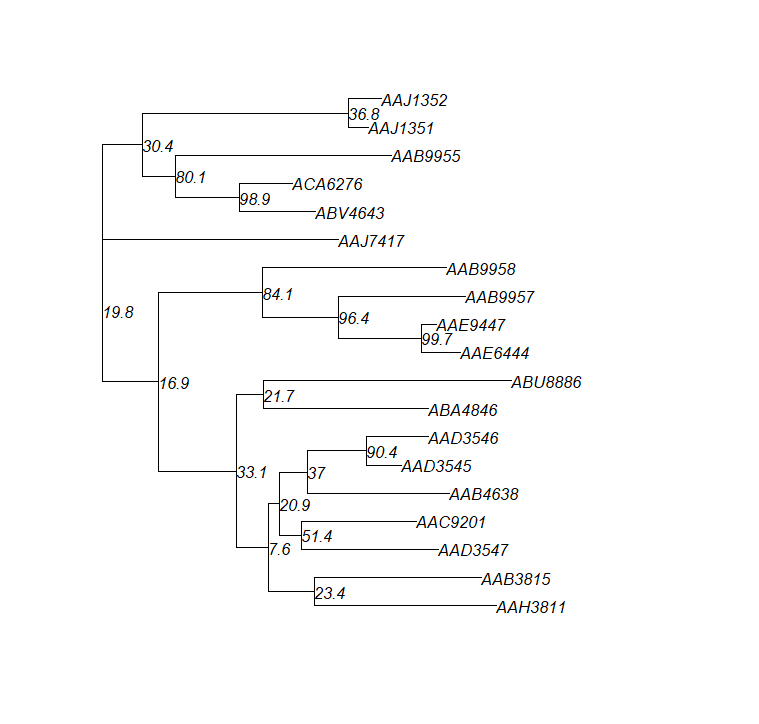


Tree showing nodal support values for *Agabus* (median = 36.8)


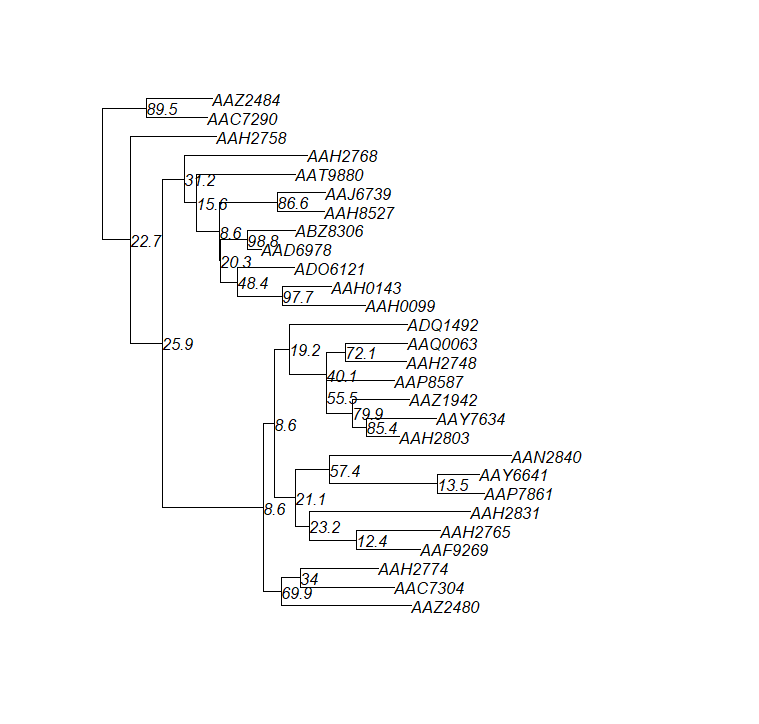


Tree showing nodal support values for *Agonum* (median = 32.6).


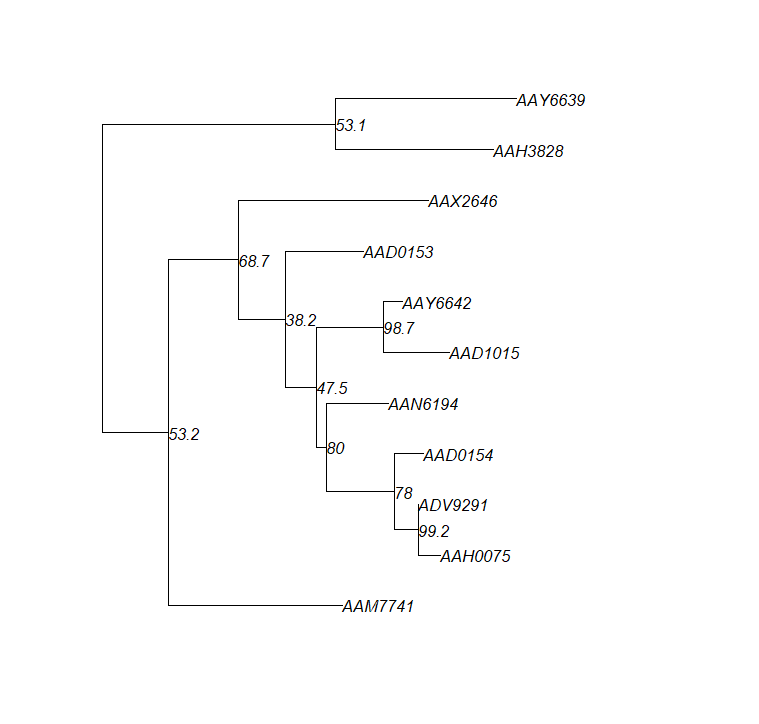


Tree showing nodal support values for *Coelambus* (median = 68.7).


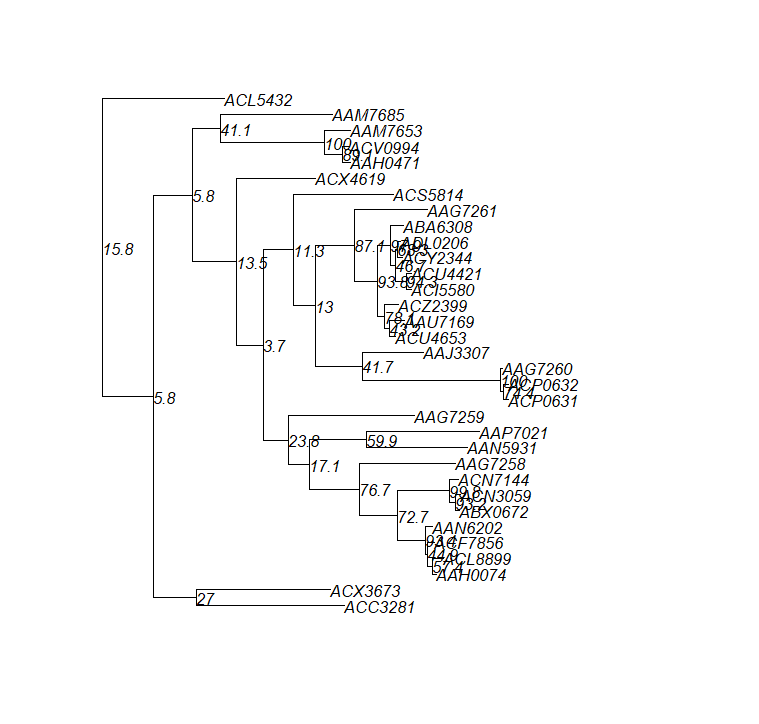


Tree showing nodal support values for *Contacyphon* (median = 58.65).


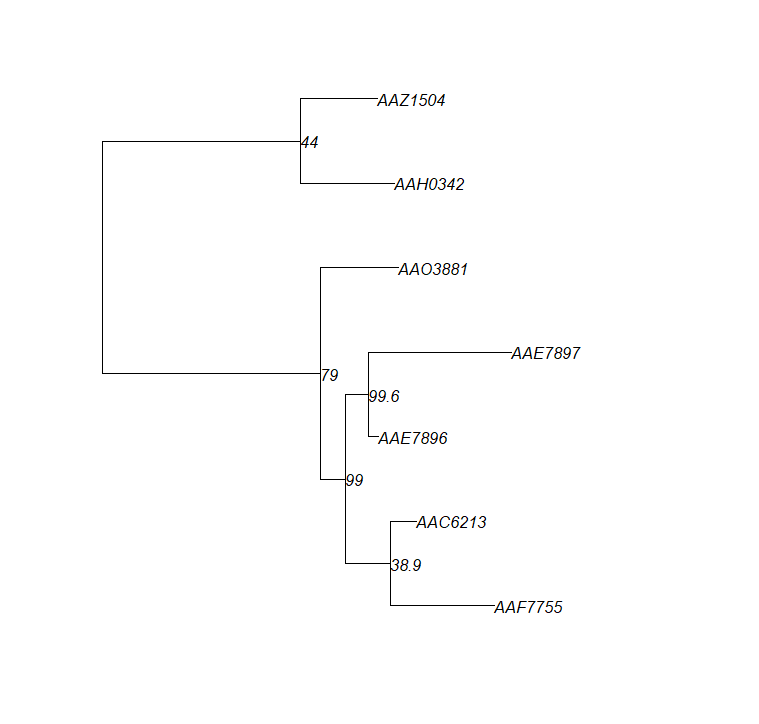


Tree showing nodal support values for *Cymindis* (median = 79).


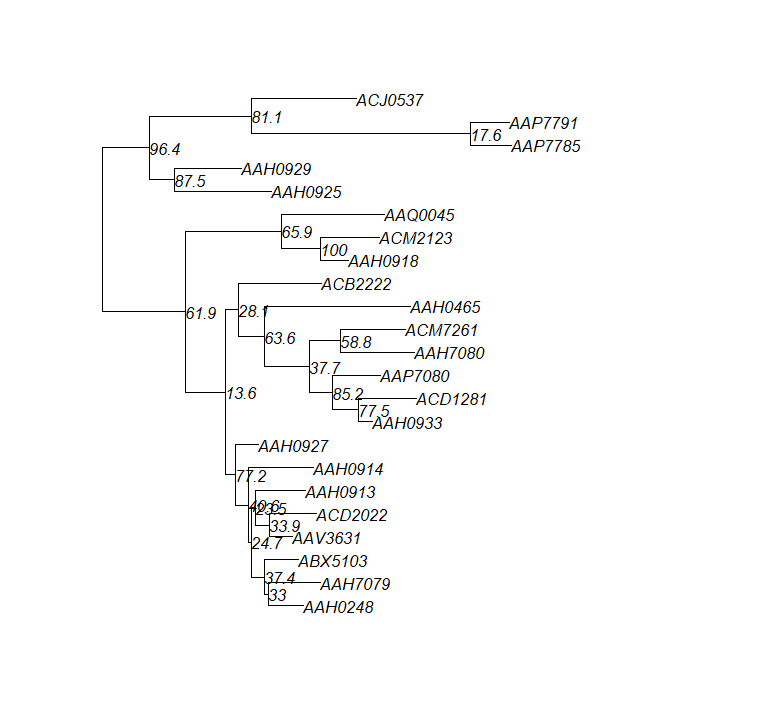


Tree showing nodal support values for *Dichelotarsus* (median = 58.8).


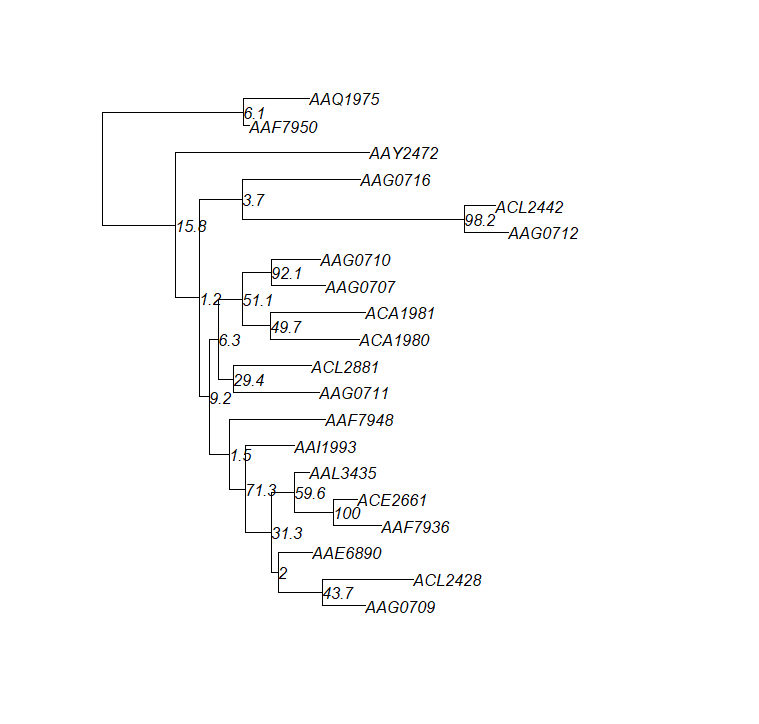


Tree showing nodal support values for *Gyrinus* (median = 30.35).


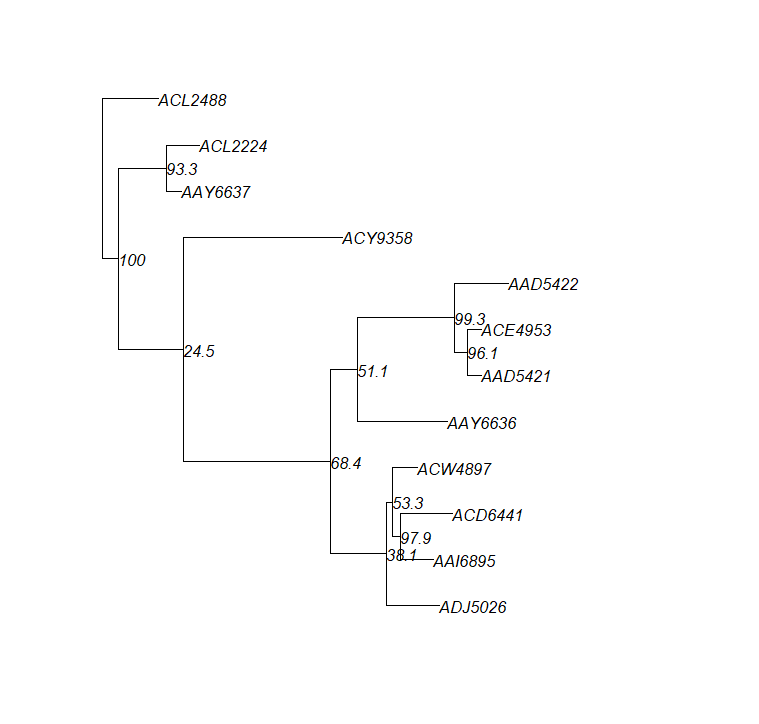


Tree showing nodal support values for *Haliplus* (median = 80.85).


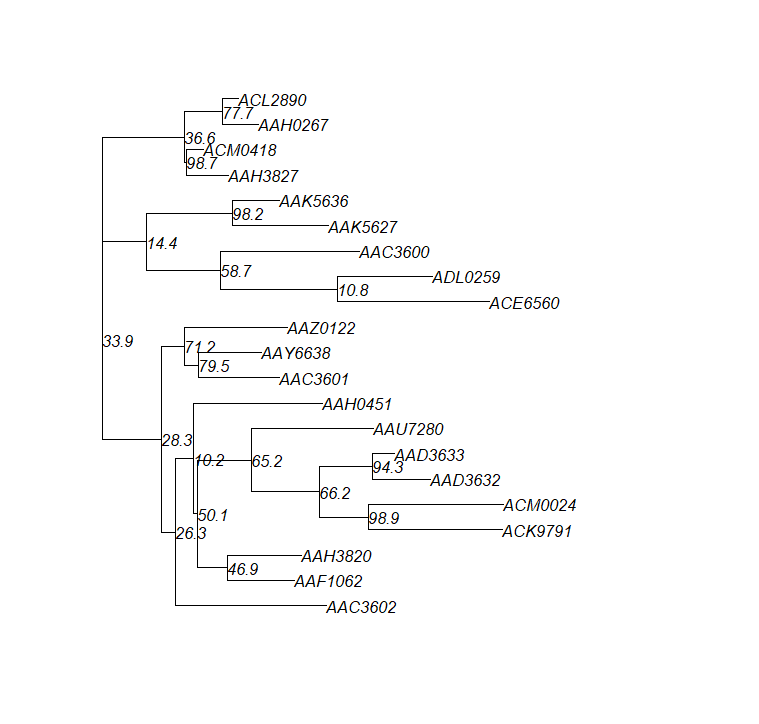


Tree showing nodal support values for *Hydroporous* (median = 58.7).


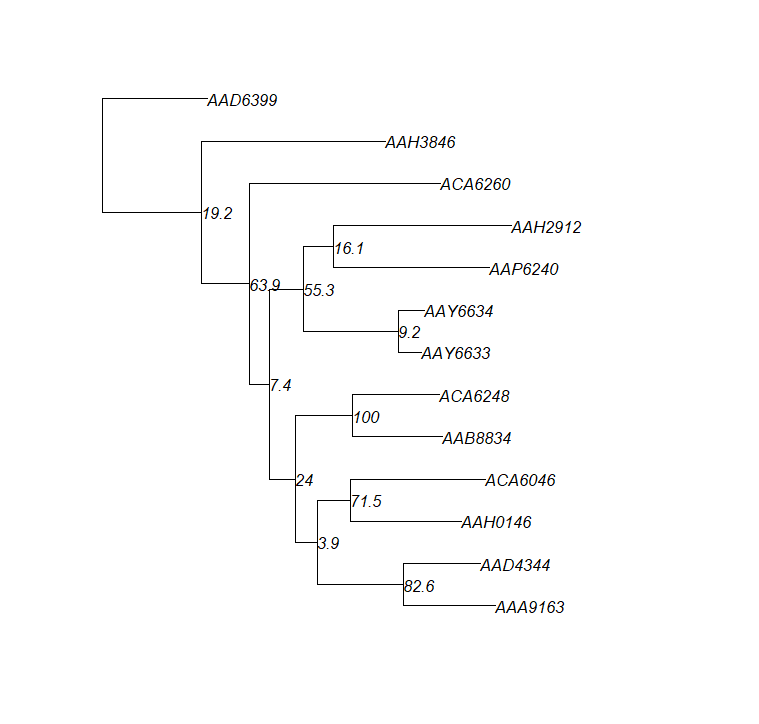


Tree showing nodal support values for *Ilybius* (median = 24).


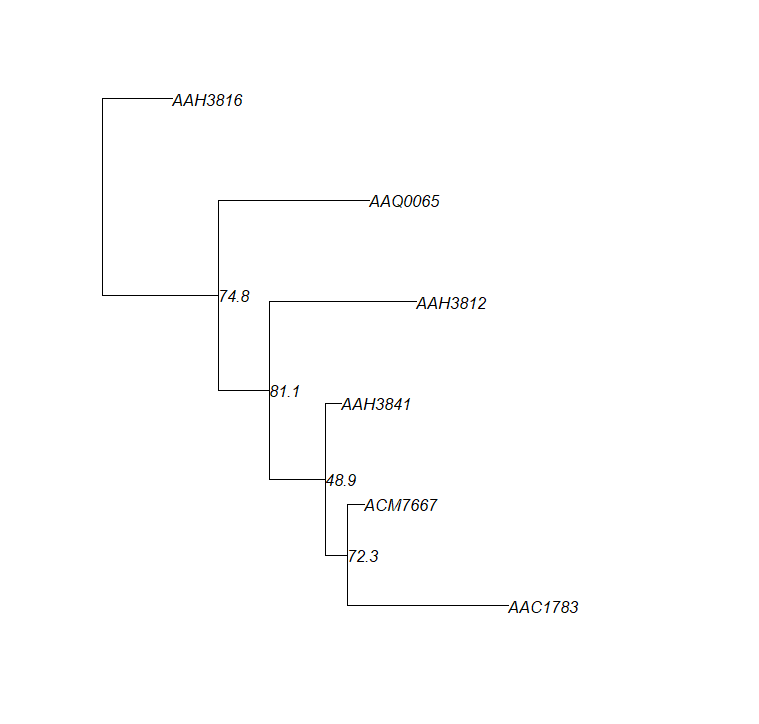


Tree showing nodal support values for *Rhantus* (median = 73.55).

Guelph Families:

Median nodal support value for Buprestidae: 47.1.

Median nodal support value for Cantharidae: 41.6.

Median nodal support value for Carabidae: 36.6.

Median nodal support value for Cerambycidae: 41.9.

Median nodal support value for Chrysomelidae: 41.1.


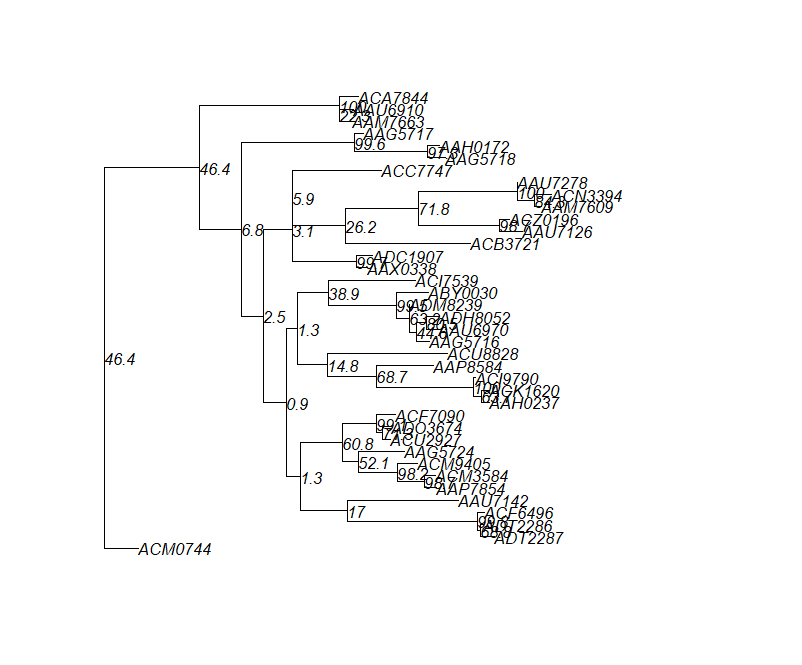


Tree showing nodal support values for Cleridae (median = 63.7).

Median nodal support value for Coccinellidae: 59.


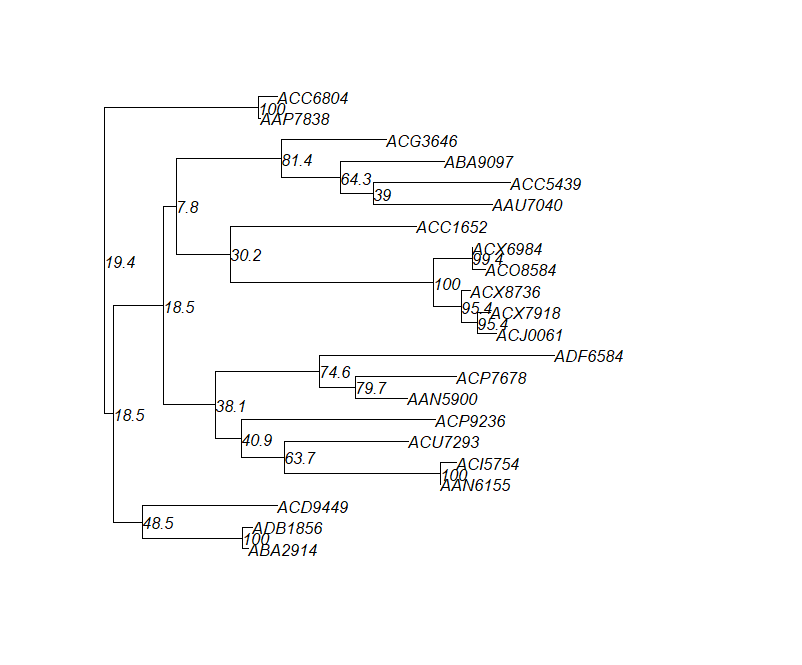


Tree showing nodal support values for Corylophidae (median = 64.3).

Median nodal support value for Cryptophagidae: 30.7.

Median nodal support value for Curculionidae: 40.2.


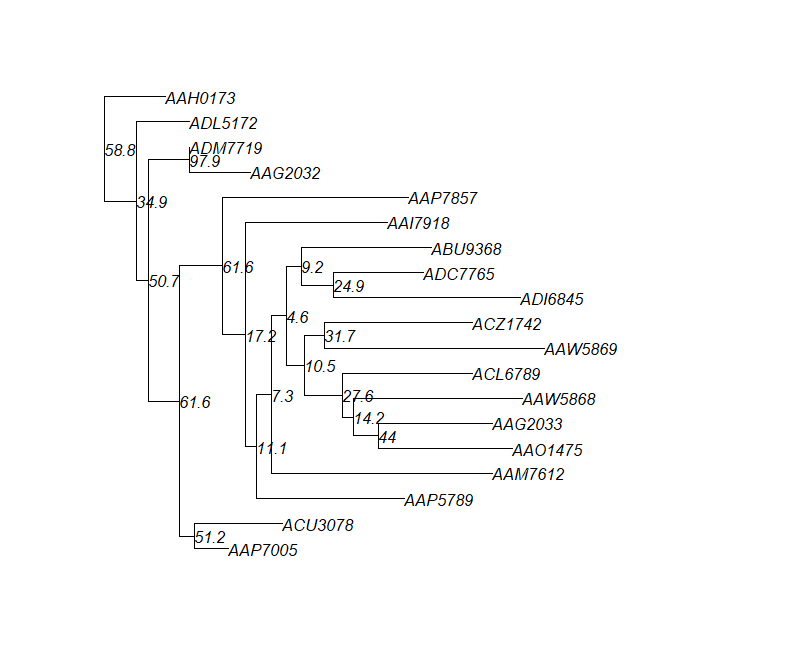


Tree showing nodal support for Dermestidae (median = 29.65).

Median nodal support for Dytiscidae: 35.7.

Median nodal support for Elateridae: 36.45.


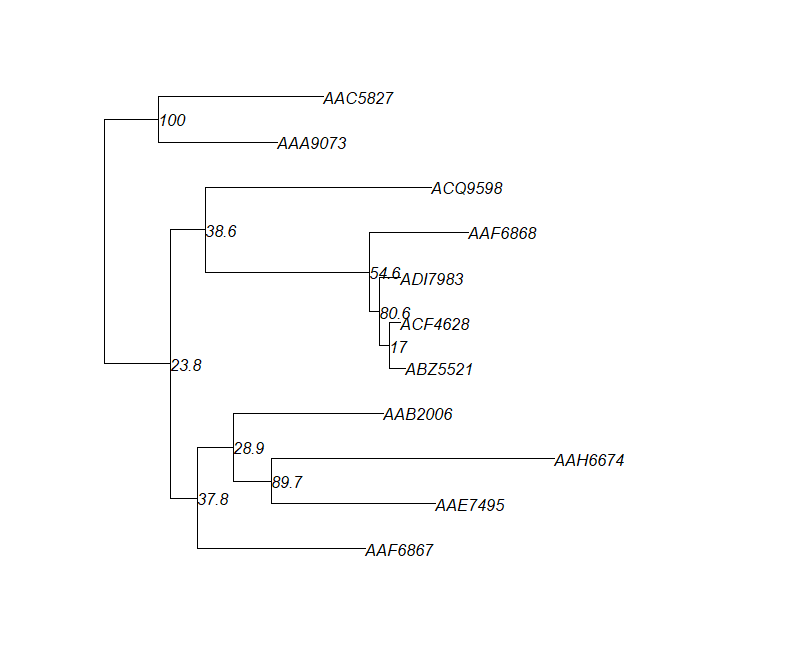


Tree showing nodal support for Elmidae (median = 38.6).


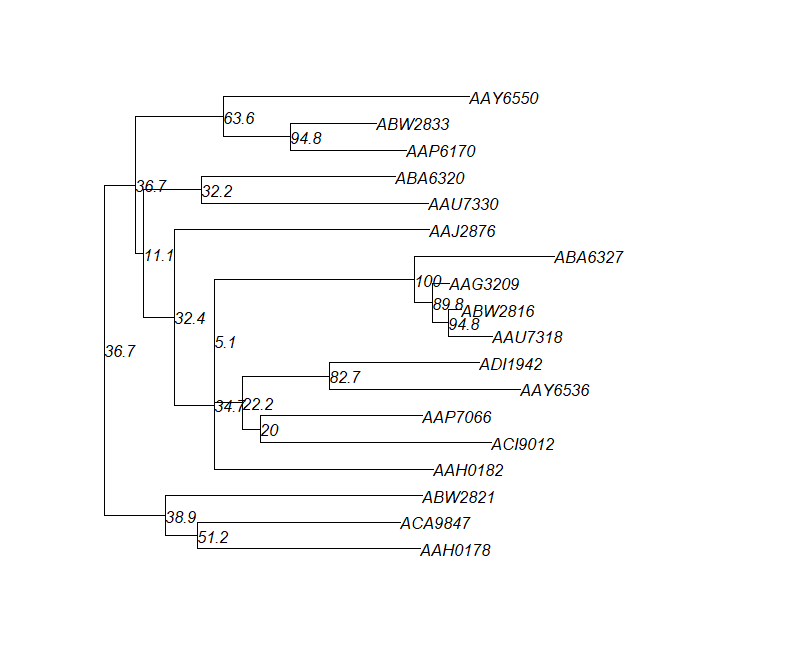


Tree showing nodal support for Erotylidae (median = 36.7).


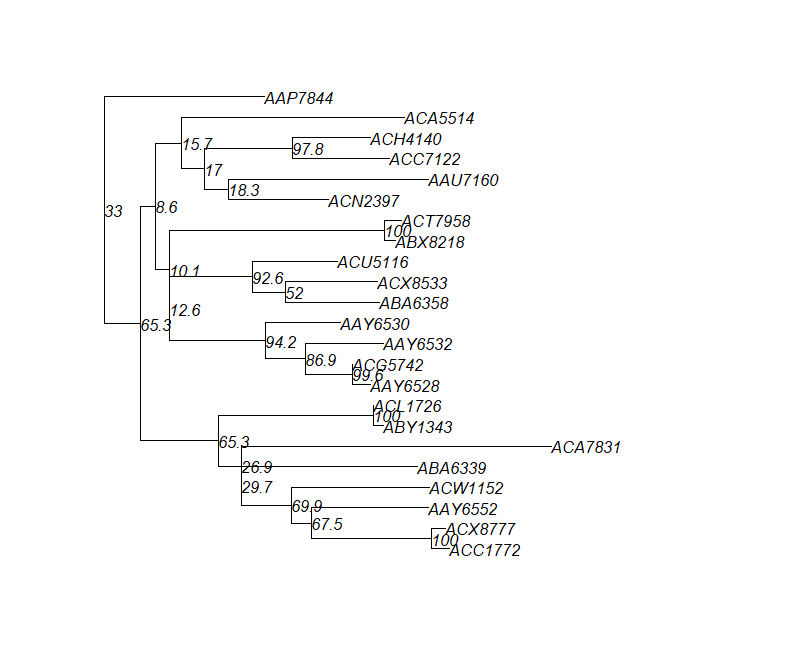


Tree showing nodal support for Eucnemidae (median = 65.3).


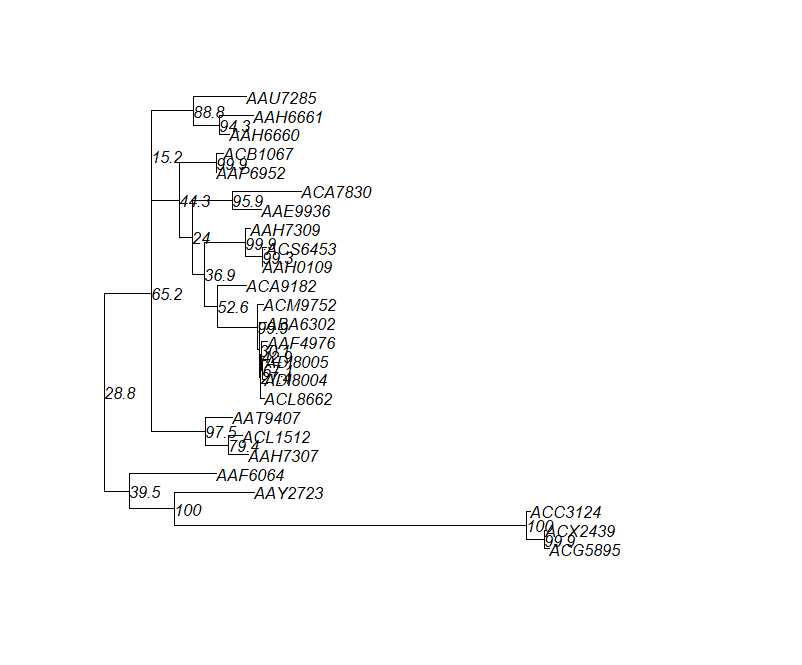


Tree showing nodal support for Lampyridae (median = 73.25)

Mean nodal support for Latridiidae: 50.4.

Mean nodal support for Leiodidae: 32.25.


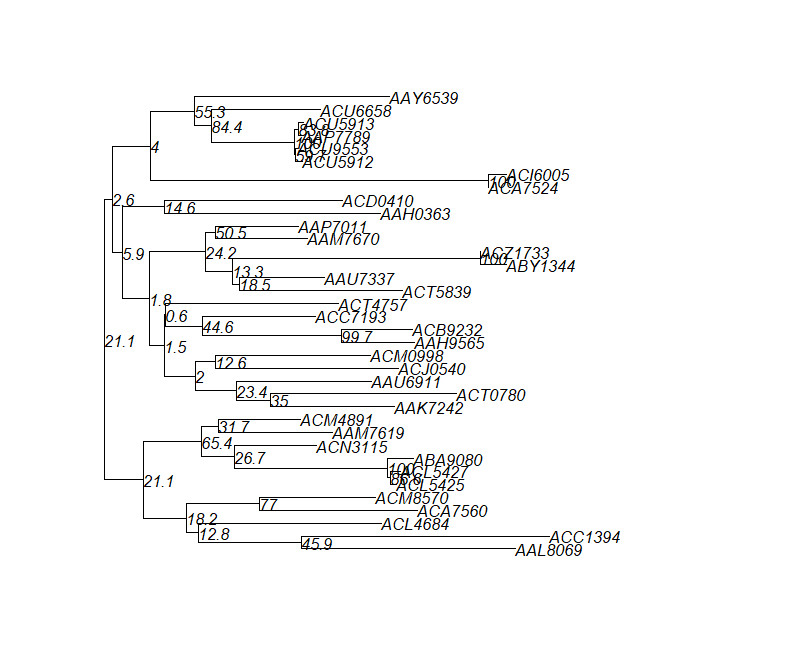


Tree showing nodal support for Melandryidae (median = 26.7).


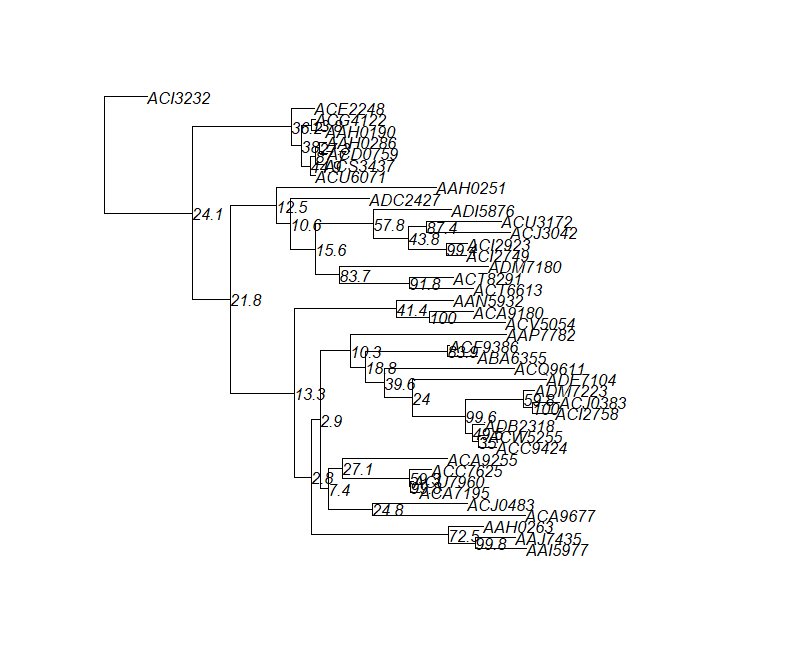


Tree showing nodal support for Melyridae (median = 39.6).

Median nodal support for Mordellidae: 46.25.


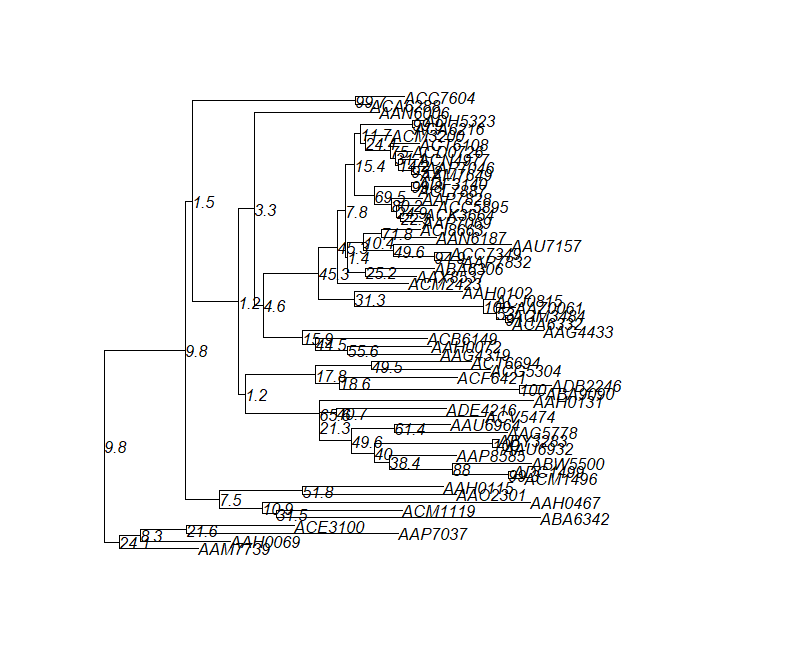


Tree showing nodal support for Nitidulidae (median = 34.95).


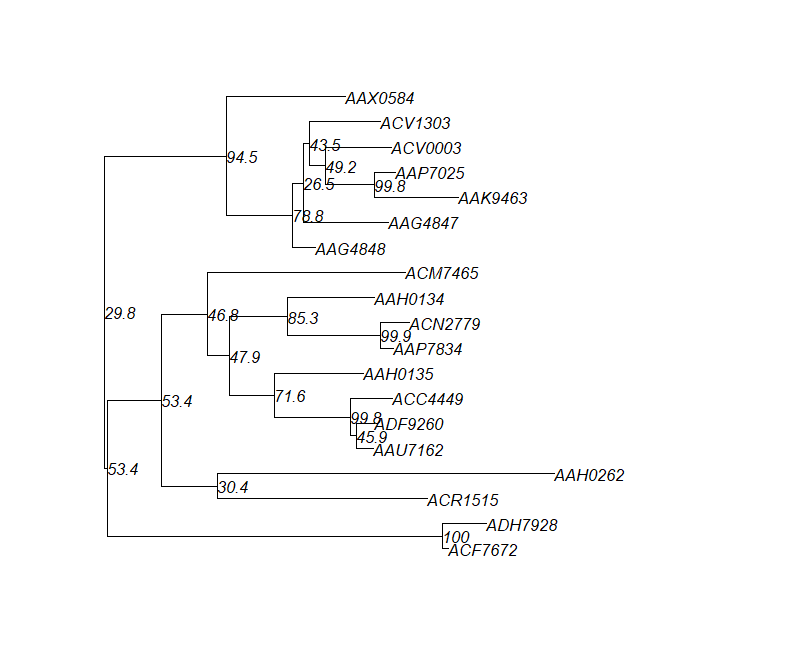


Tree showing nodal support for Phalacridae (median = 53.4).


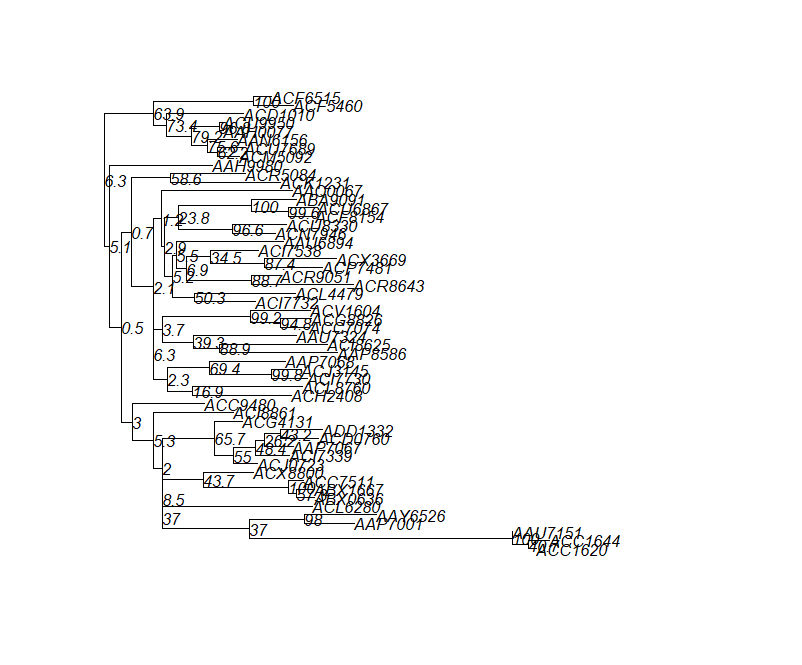


Tree showing nodal support for Ptinidae (median = 43.7).


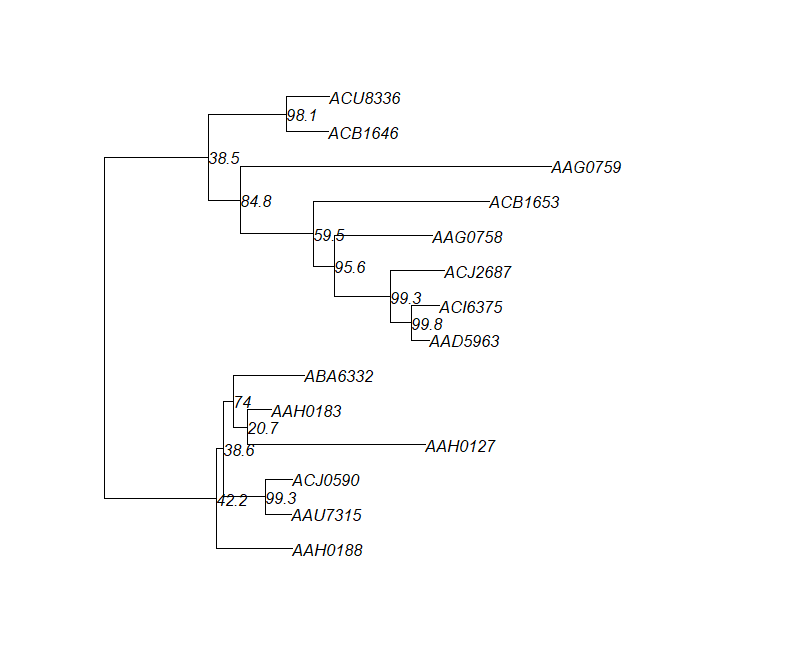


Tree showing nodal support for Pyrochroidae (median = 79.4).

Median nodal support for Scarabaeidae: 57.2.


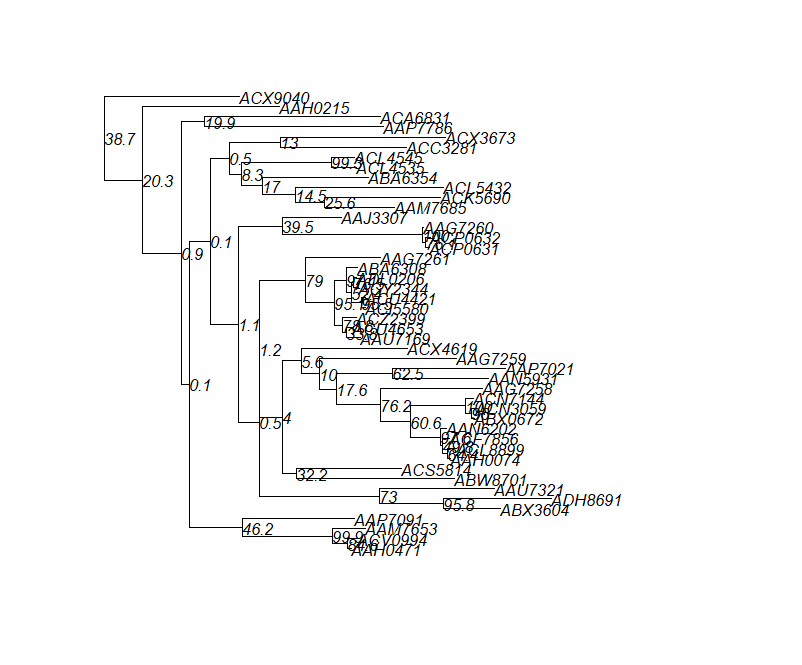


Tree showing nodal support for Scirtidae (median = 46.2).


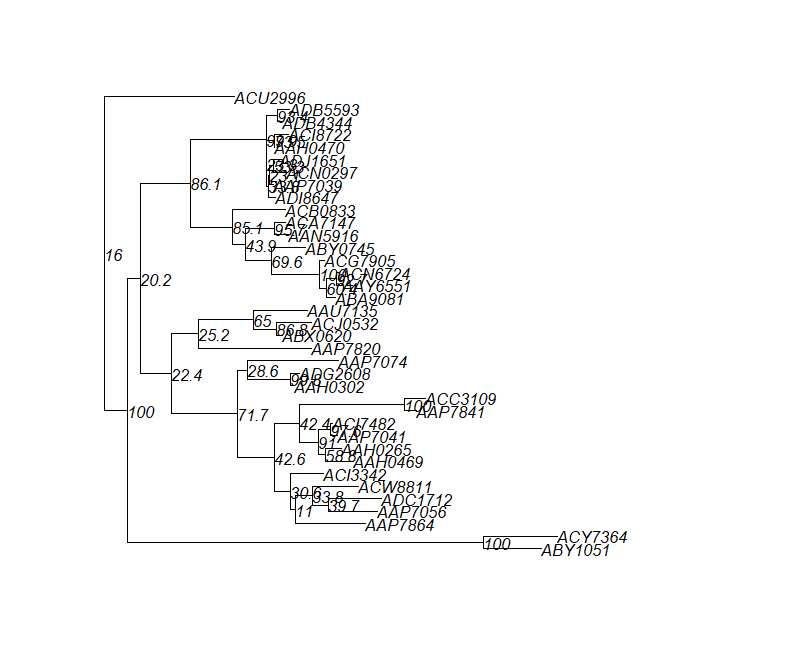


Tree showing nodal support for Scraptiidae (median = 62.7).

Median nodal support for Staphylinidae: 24.3.


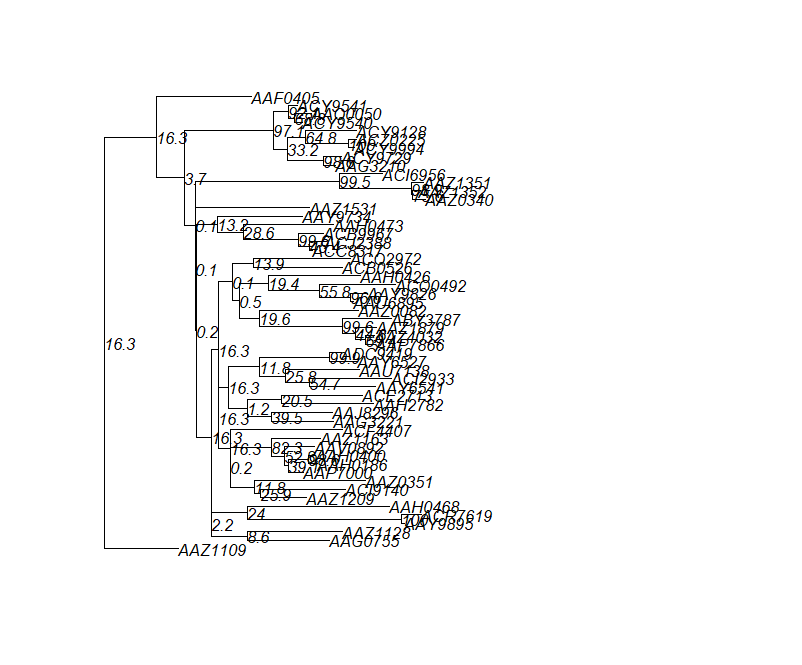


Tree showing nodal support for Tenebrionidae (median = 25.9).


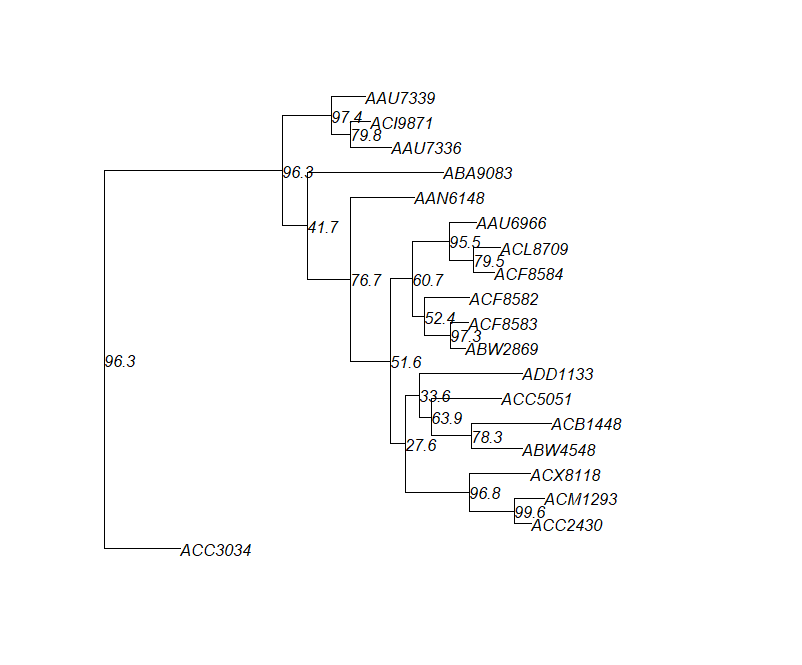


Tree showing nodal support for Throscidae (median = 78.9).

Guelph Genera:


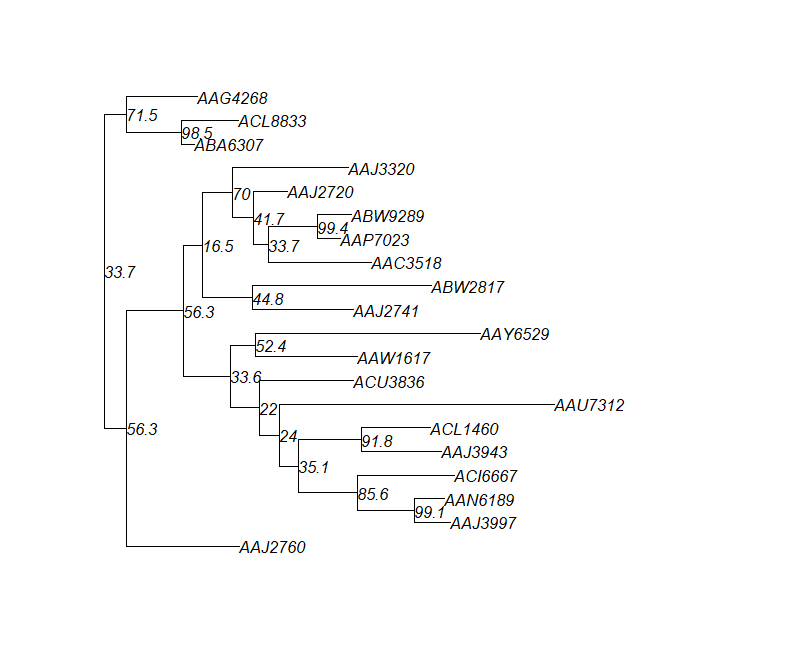


Tree showing nodal support for *Aleochara* (median = 52.4).


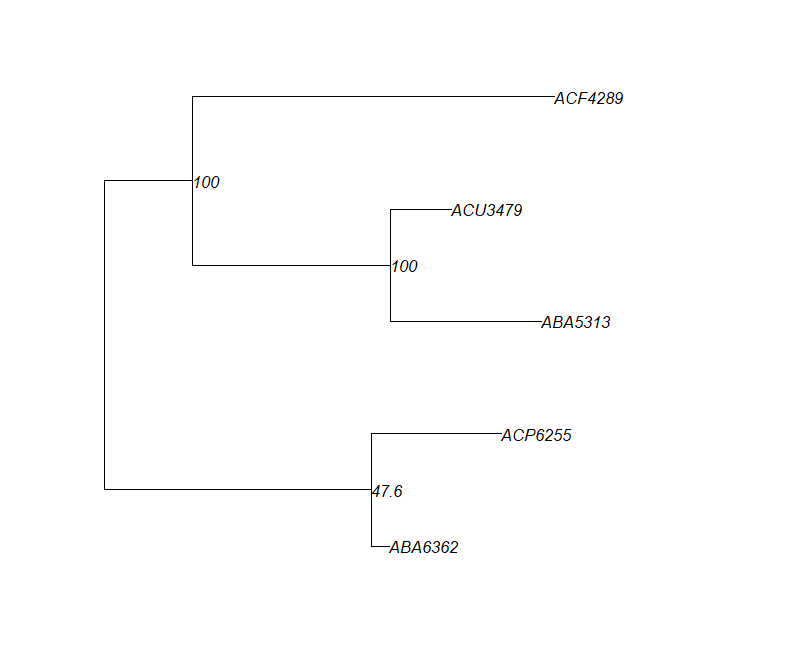


Tree showing nodal support for *Amischa* (median = 100).


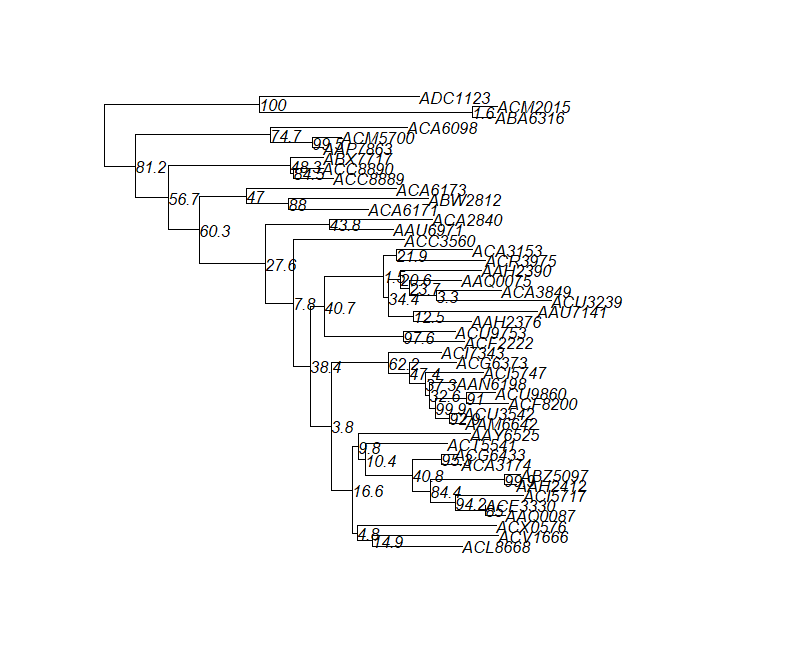


Tree showing nodal support for *Ampedus* (median = 43.8).


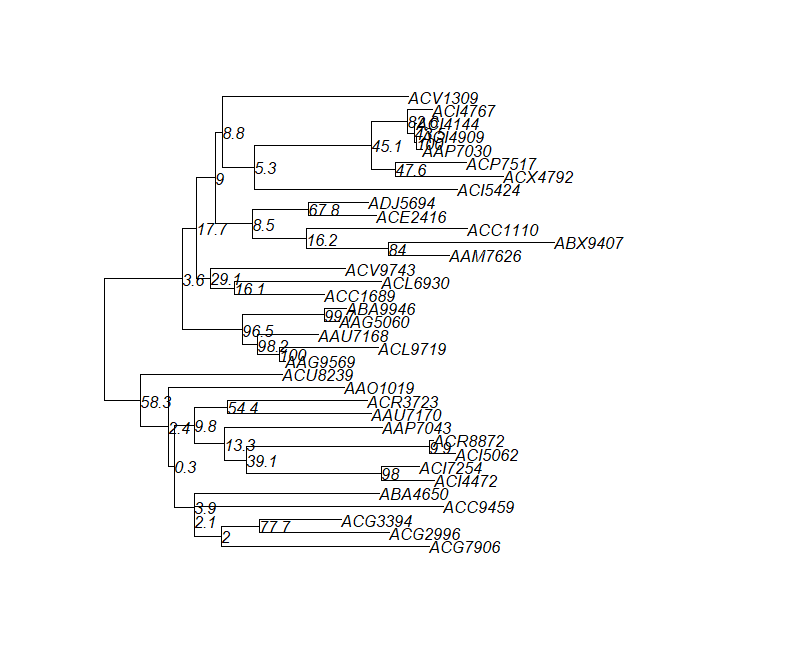


Tree showing nodal support for *Atomaria* (median = 29.1).


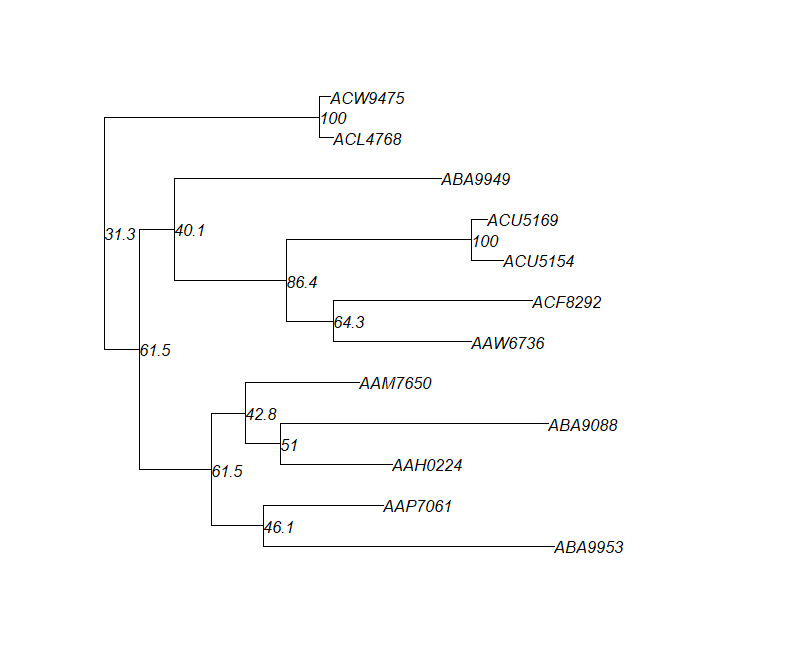


Two showing nodal support for *Chaetocnema* (median = 61.5).


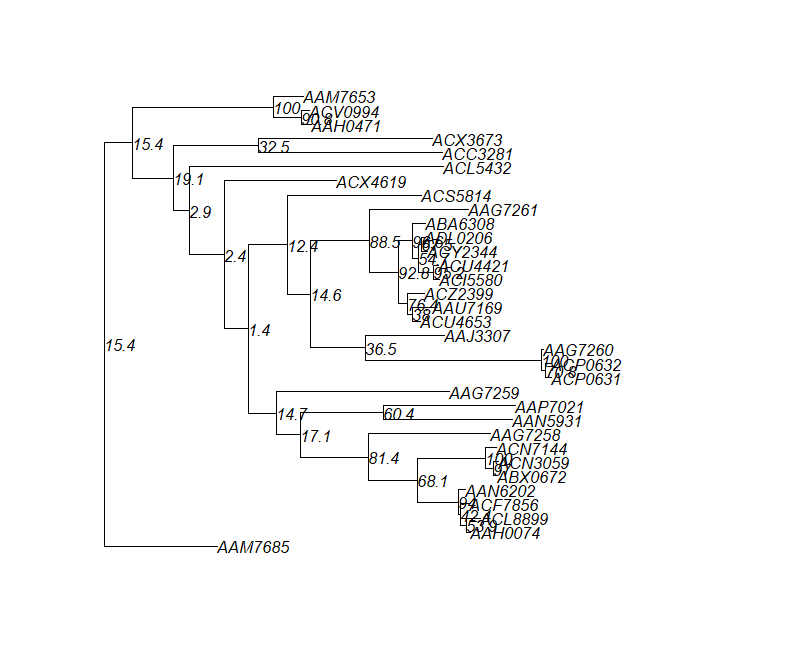


Tree showing nodal support for *Contacyphon* (median = 57.55).


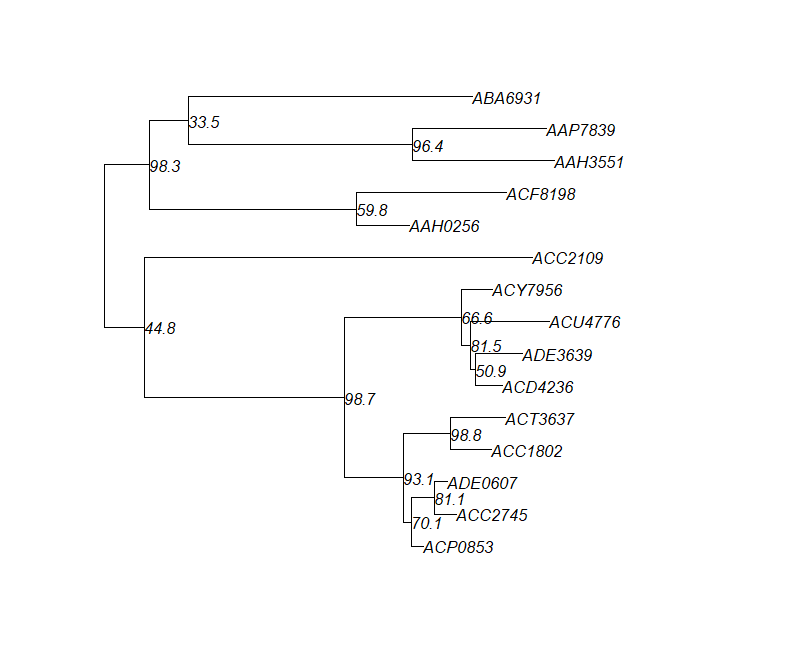


Tree showing nodal support for *Corticarina* (median = 81.1).


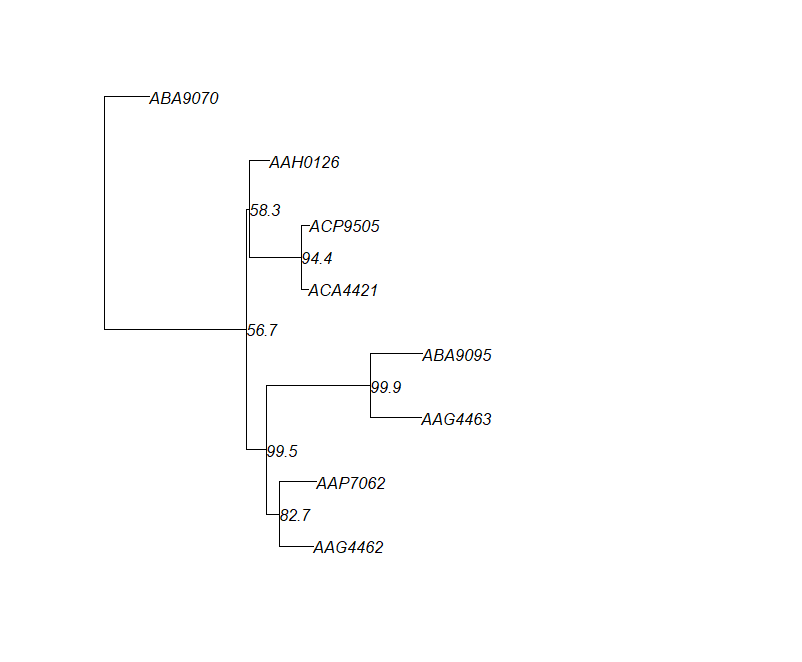


Tree showing nodal support for *Crepidodera* (median = 88.55).


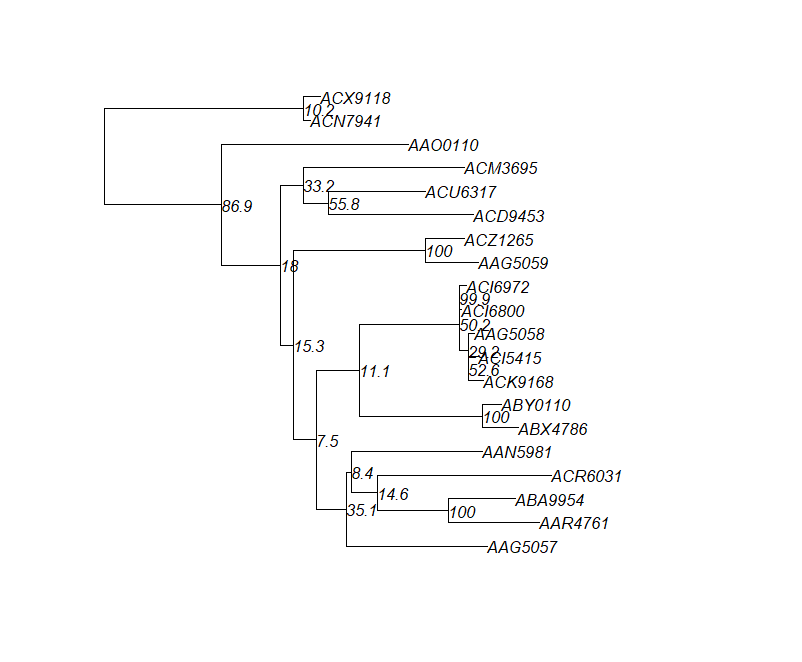


Tree showing nodal support for *Cryptophagus* (median = 34.15).


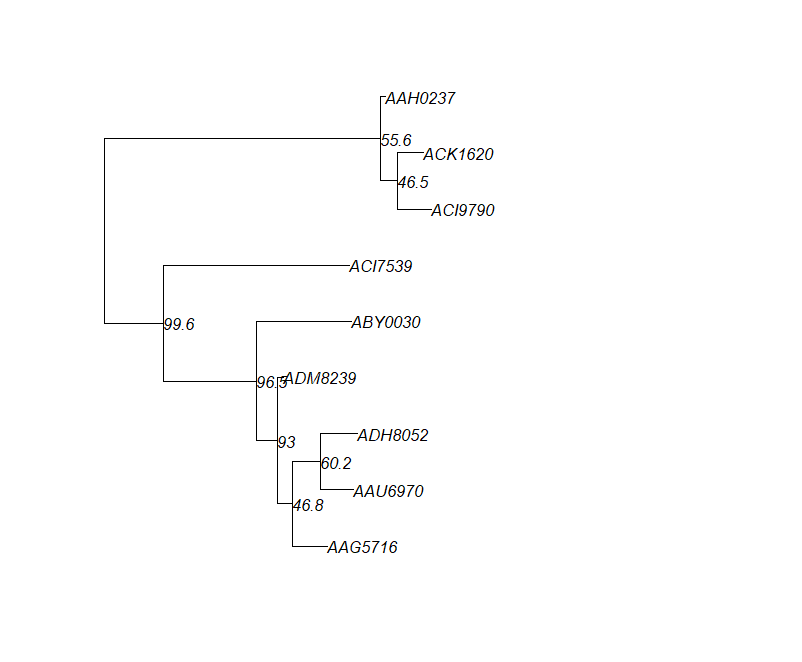


Tree showing nodal support for *Enoclerus* (median = 60.2).


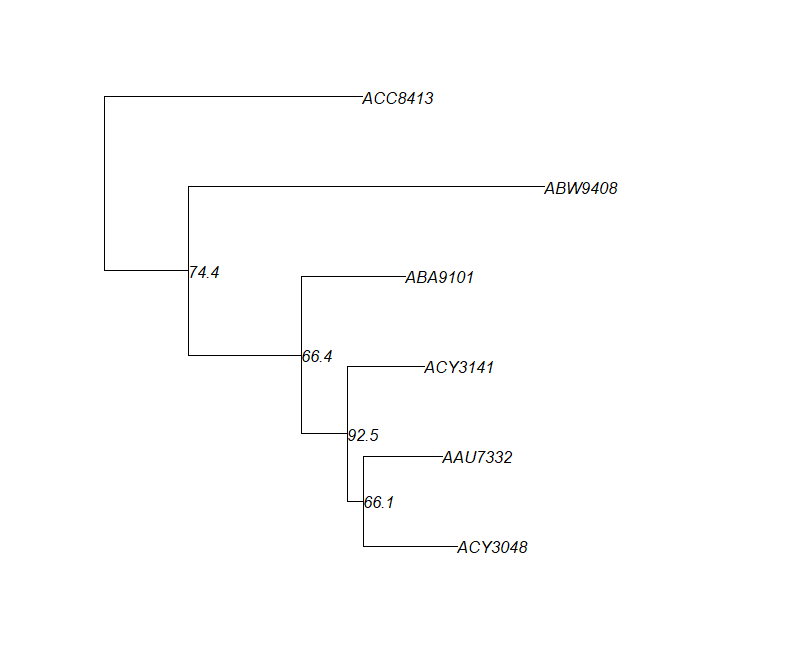


Tree showing nodal support for *Epitrix* (median = 70.4).


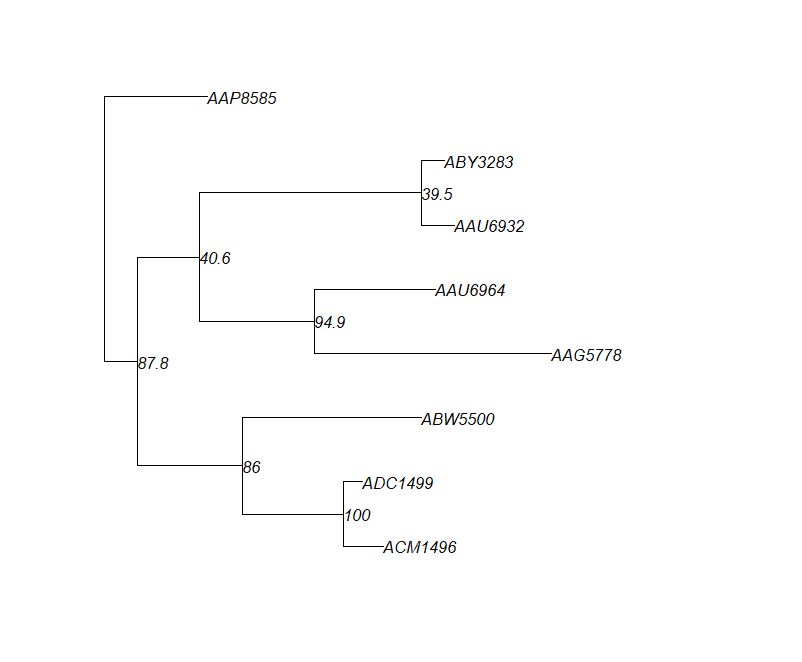


Tree showing nodal support for *Glischrochilus* (median = 86.9).


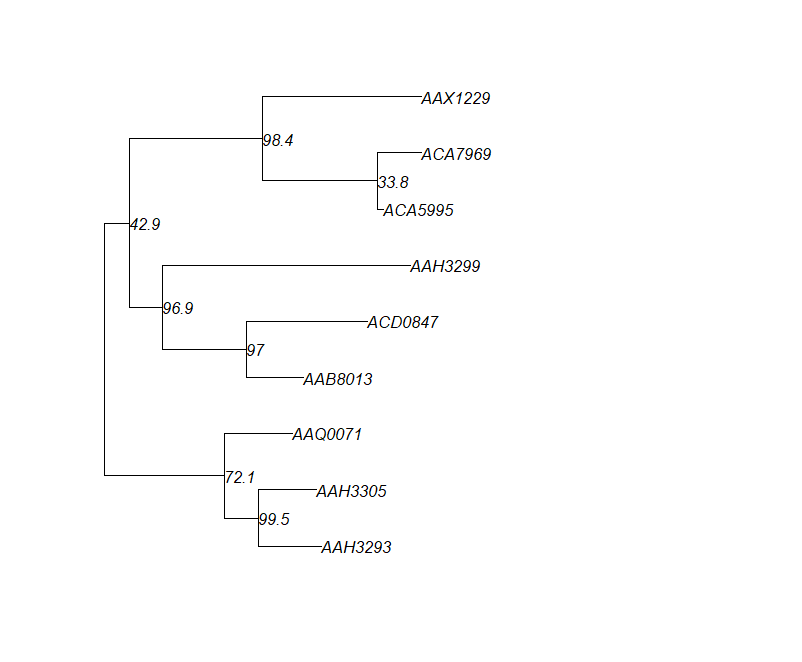


Tree showing nodal support for *Hippodamia* (median = 96.9).


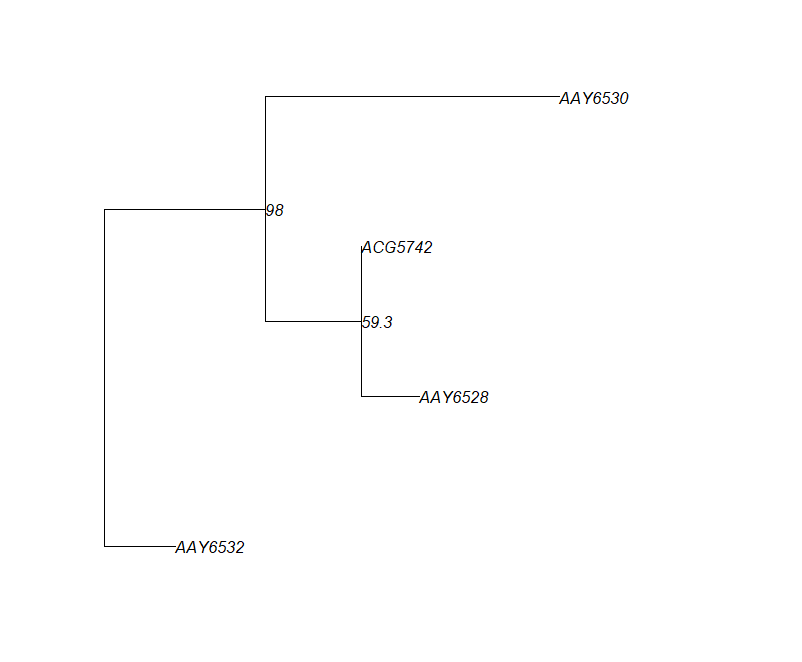


Tree showing nodal support for *Isorhipis* (median = 78.65).


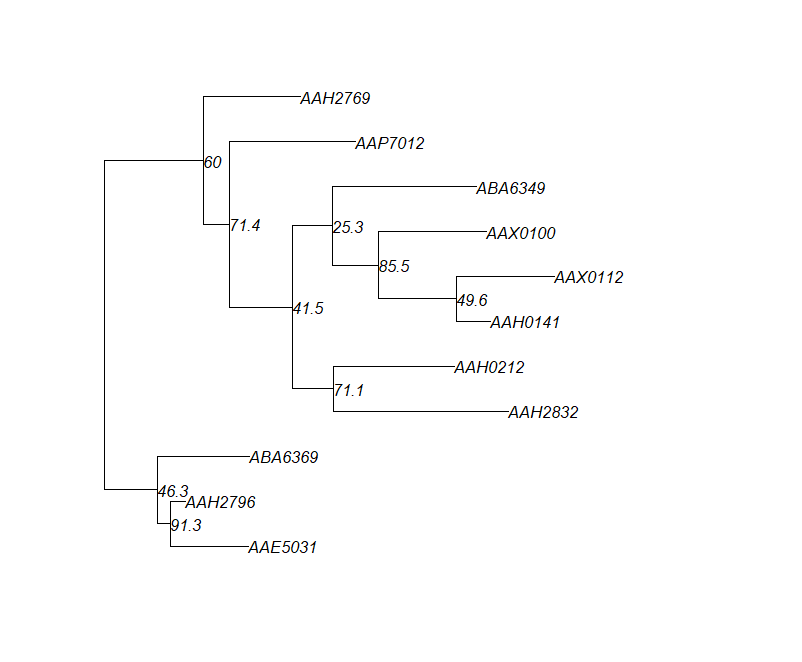


Tree showing nodal support for *Lebia* (median = 60).


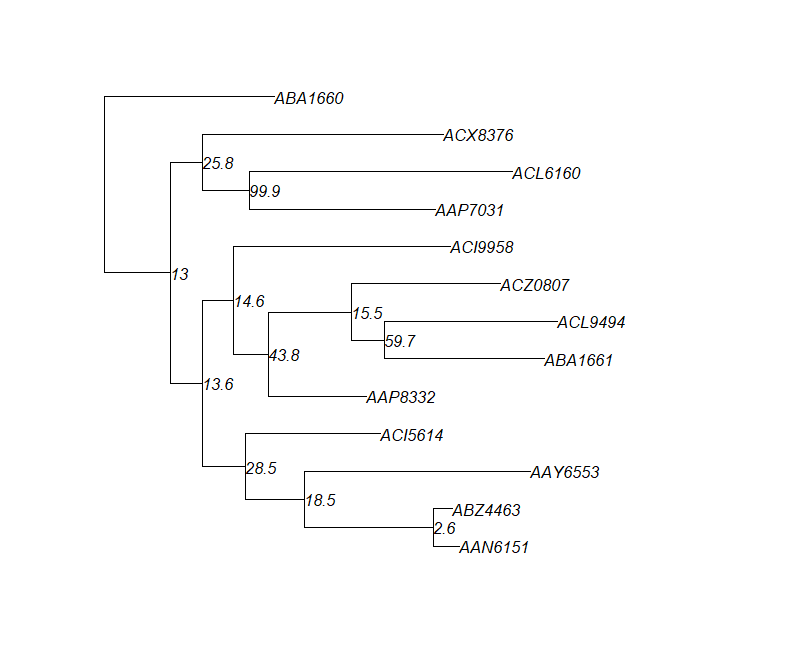


Tree showing nodal support for *Longitarsus* (median = 18.5).


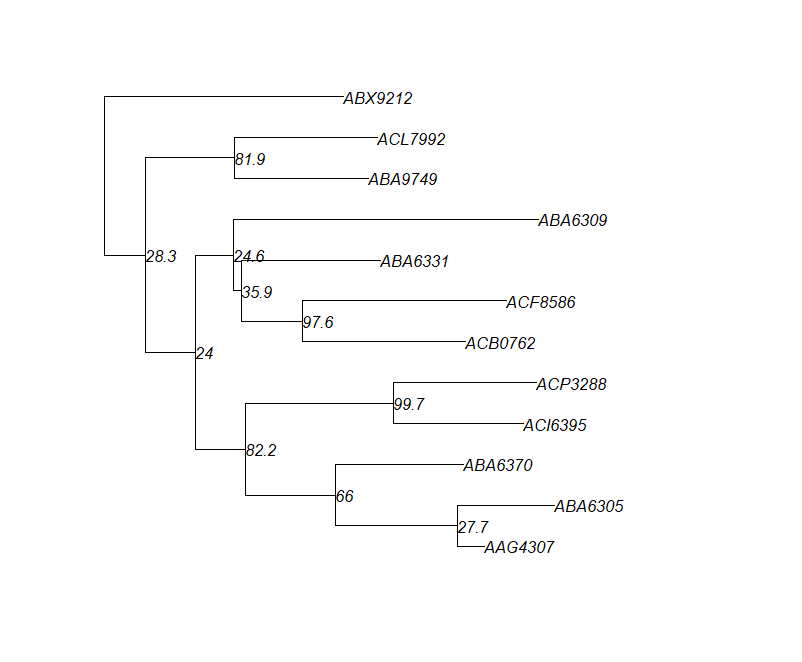


Tree showing nodal support for *Lordithon* (median = 50.95).


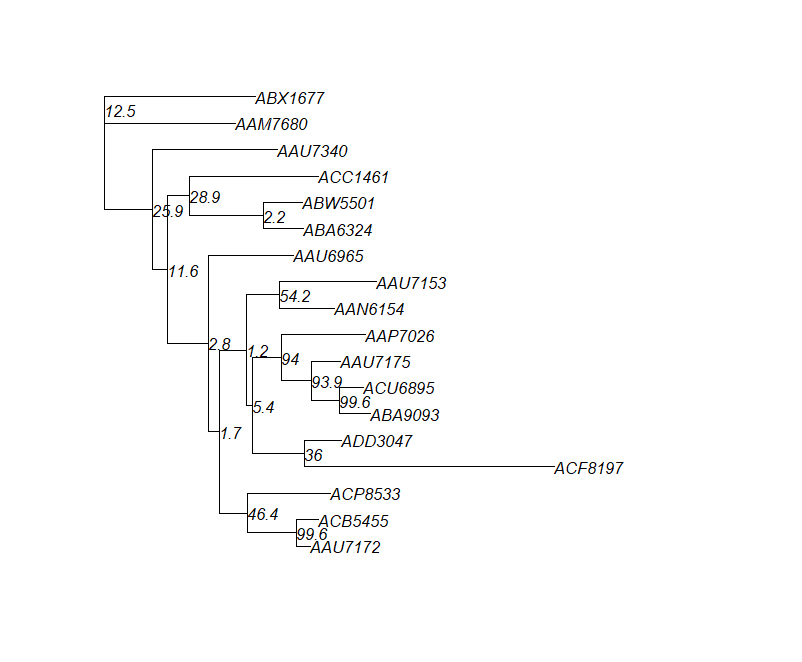


Tree showing nodal support for *Melanophthalma* (median = 27.4).


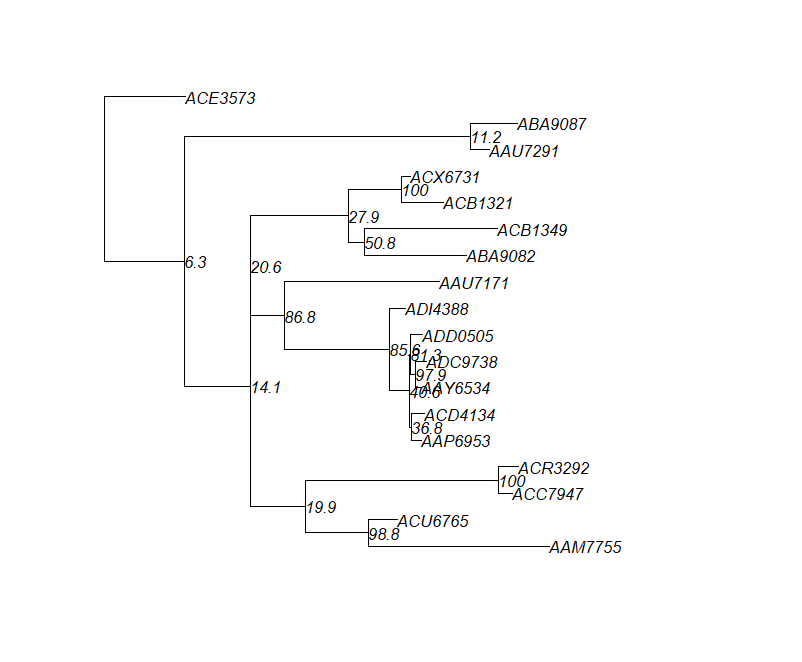


Tree showing nodal support for *Mordellina* (median = 45.7).


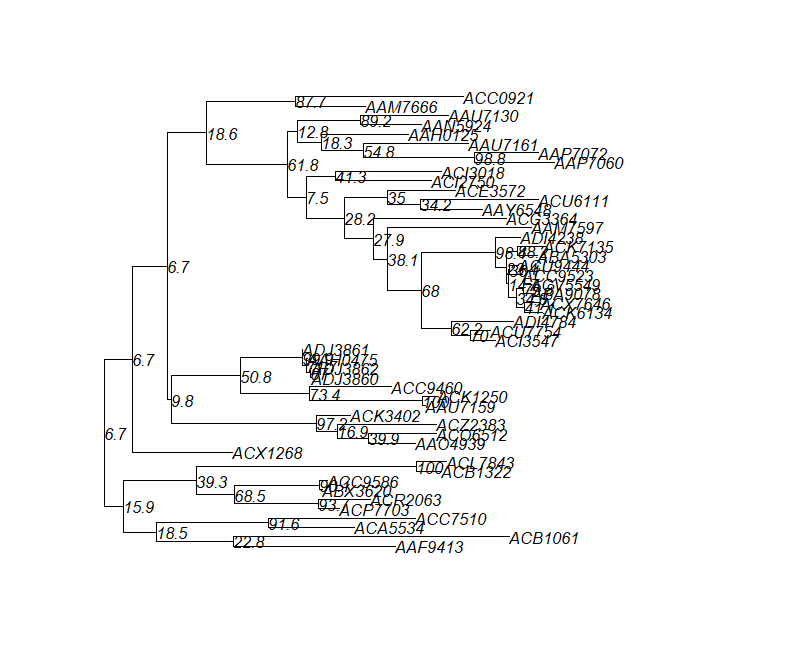


Tree showing nodal support for *Mordellistena* (median = 41.2).


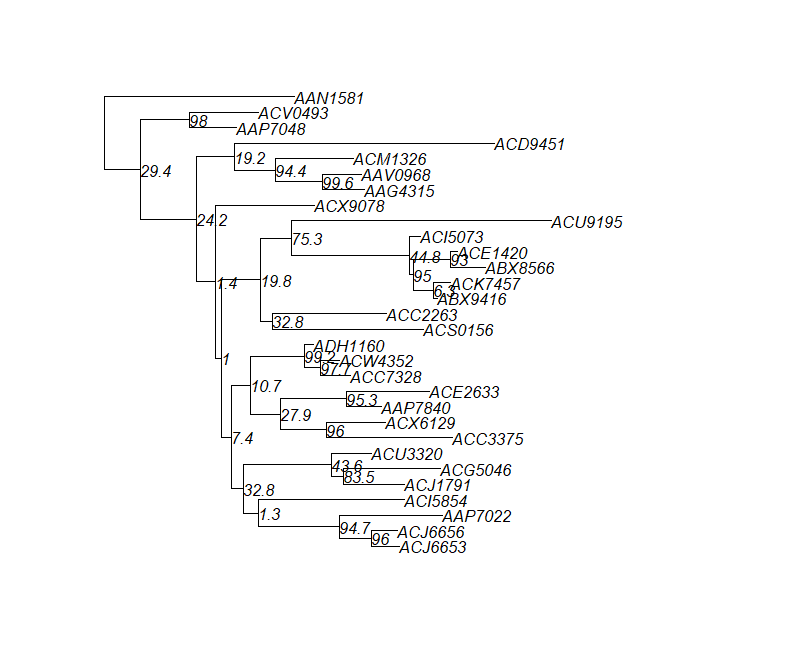


Tree showing nodal support for *Oxypoda* (median = 44.2).


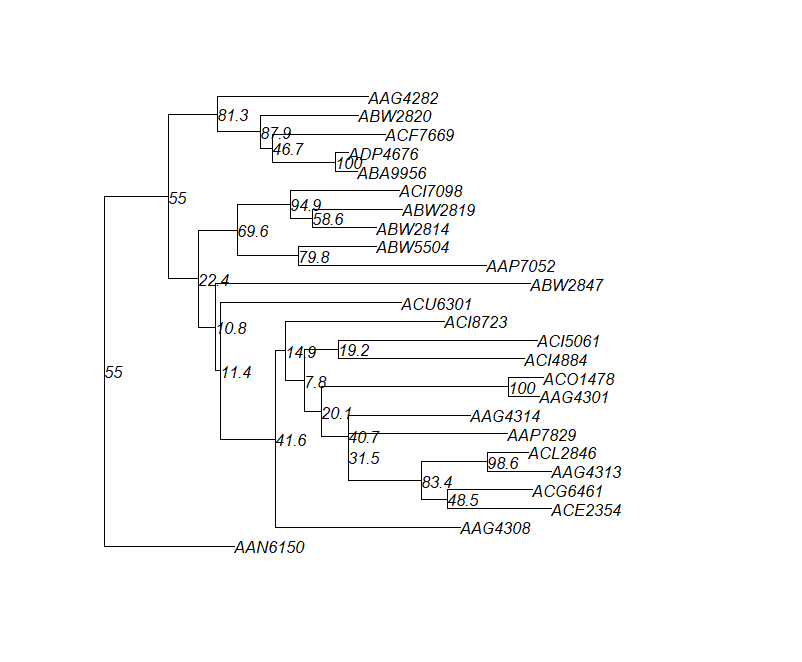


Tree showing nodal support for *Philhygra* (median = 51.75).


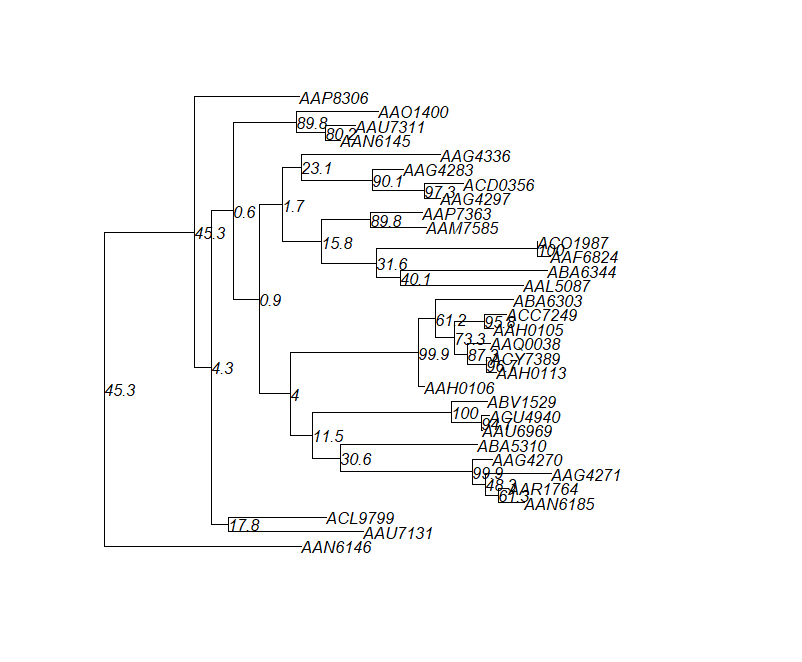


Tree showing nodal support for *Philonthus* (median = 61.2).


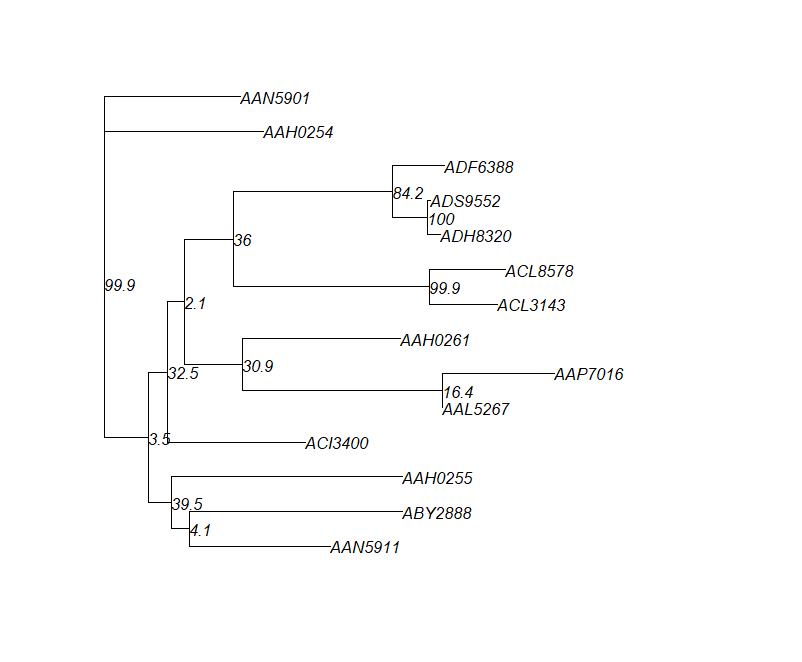


Tree showing nodal support for *Phyllotreta* (median = 34.25).


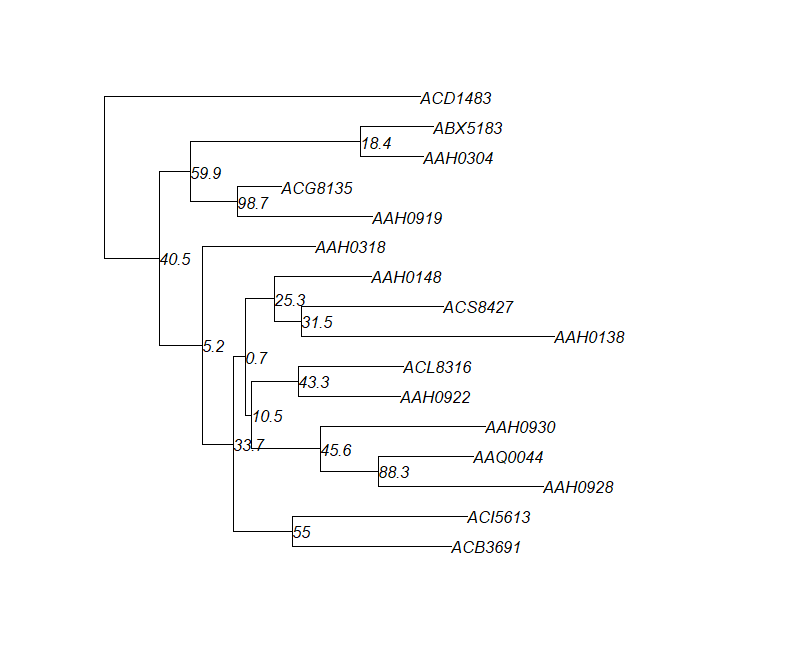


Tree showing nodal support for *Podabrus* (median = 37.1).


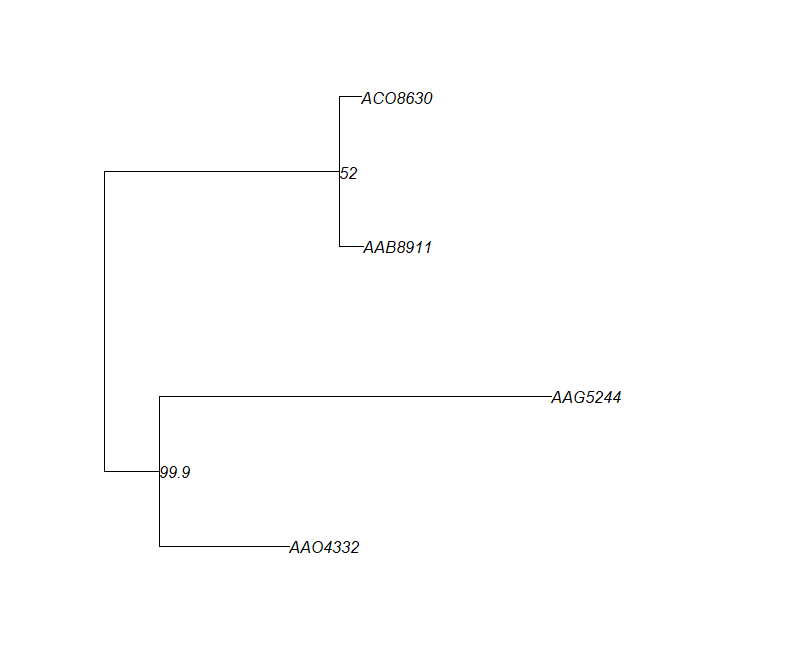


Tree showing nodal support for *Polydrusus* (median = 75.95).


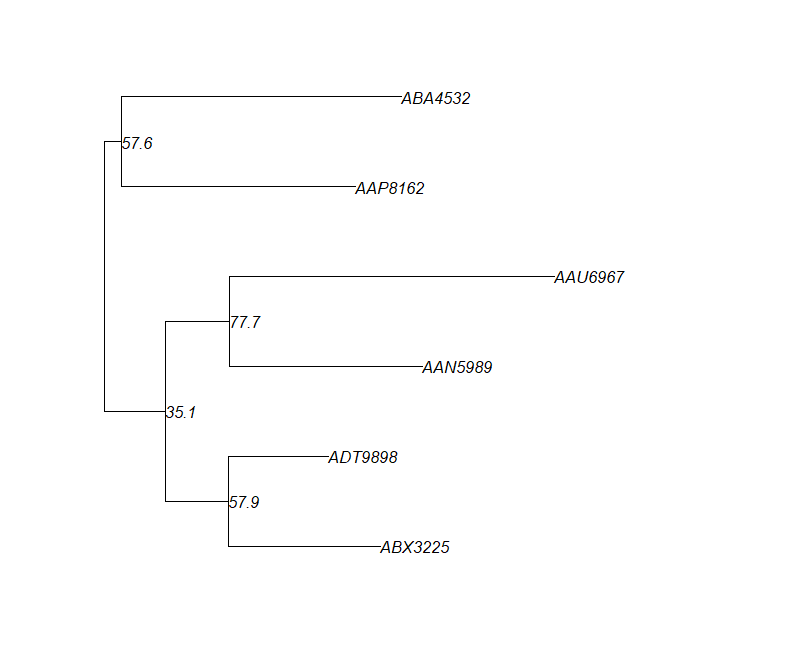


Tree showing nodal support for *Psylliodes* (median = 57.75).


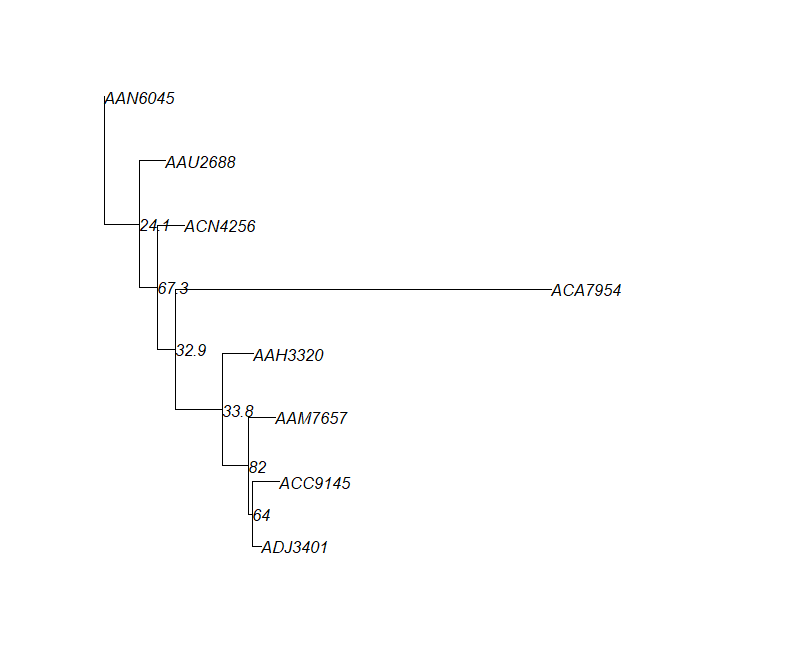


Tree showing nodal support for *Psyllobora* (median = 48.9).


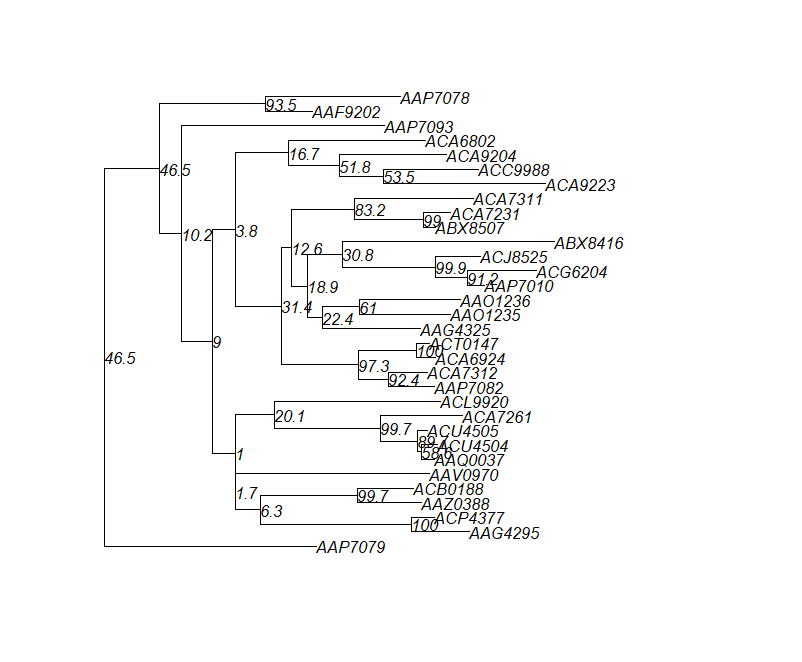


Tree showing nodal support for *Quedius* (median = 51.8).


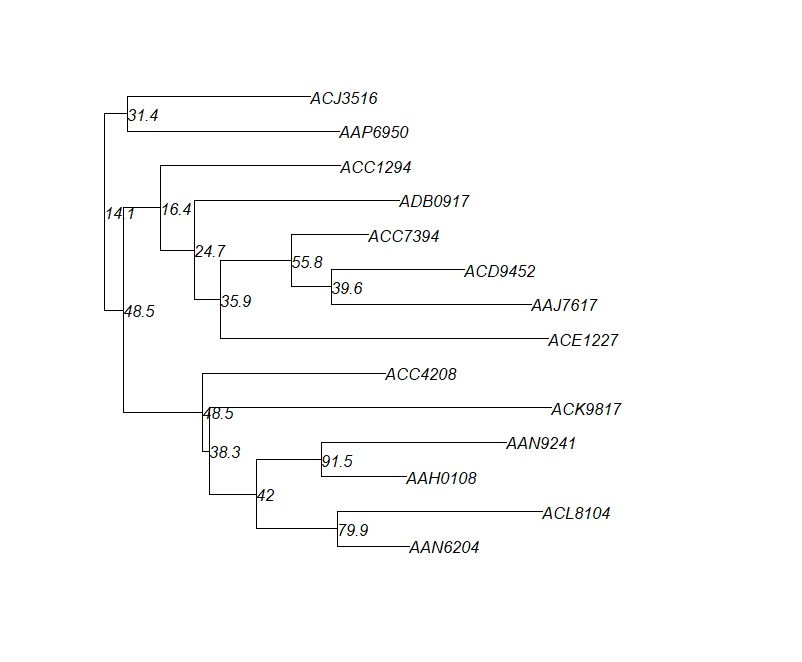


Tree showing nodal support for *Sepedophilus* (median = 39.6).


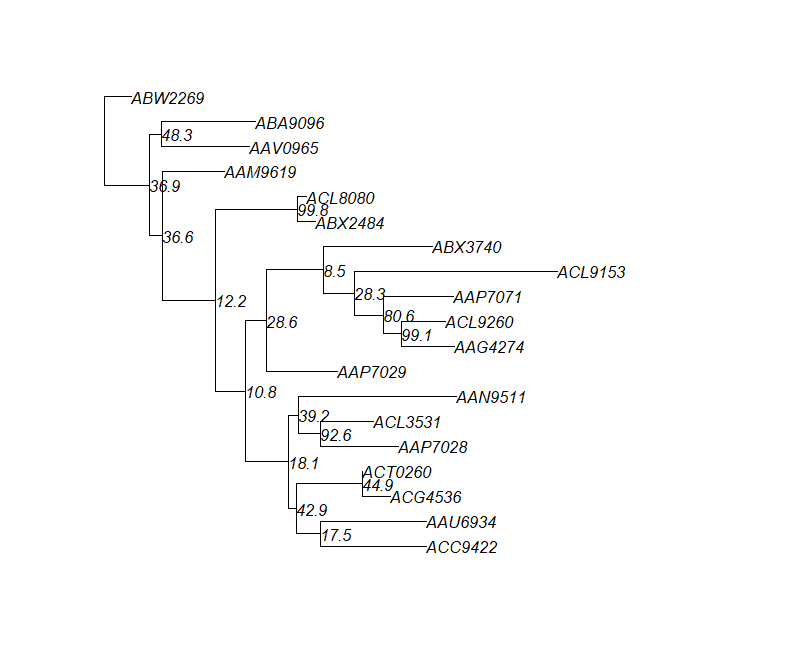


Tree showing nodal support for *Tachyporus* (median = 36.9).


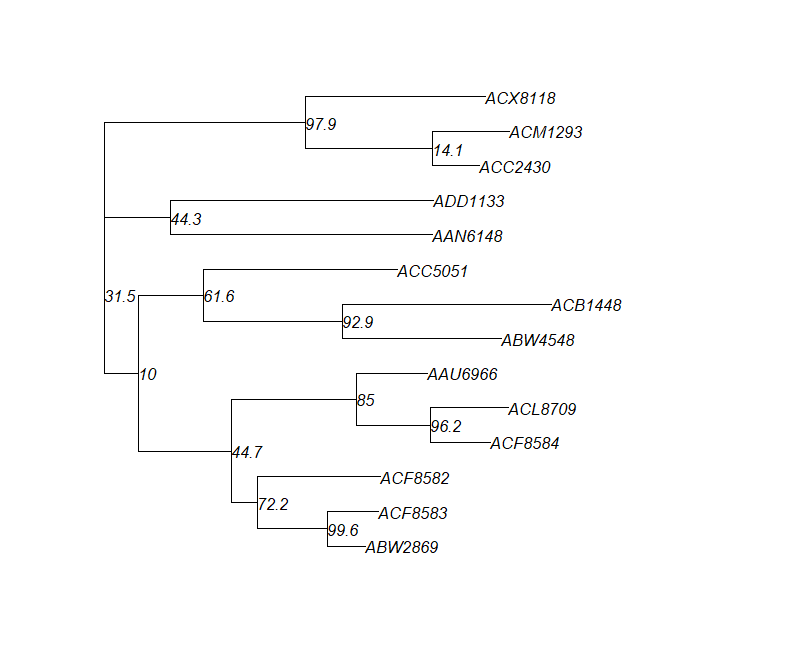


Tree showing nodal support for *Trixagus* (median = 66.9).

Appendix 2: Consensus Tree Results

Table 1: Table showing the results from the analysis using a maximum clade credibility tree. These results did not significantly differ from the original analysis.

| Family | Number of BINs in Canada and Alaska | Number of BINs in Churchill | % of Total  Found in Churchill | Number of Sequences in Canada and Alaska | Number of Sequences in Churchill | Habitat | Adult Feeding Mode | Larval Feeding Mode | Clustering Value NRI | p-Value NRI | Clustering Value NTI | p-Value NTI |
| --- | --- | --- | --- | --- | --- | --- | --- | --- | --- | --- | --- | --- |
| Buprestidae | 87 | 3 | 4% | 470 | 3 | Terrestrial | Phyto | Phyto | 1.61 | 0.07 | 2.01 | 0.07 |
| Cantharidae | 101 | 6 | 6% | 5043 | 23 | Terrestrial | Pred | Pred | ***2.45*** | ***0.002*** | **2.44** | **0.007** |
| Carabidae | 418 | 20 | 5% | 3642 | 90 | Terrestrial | Pred | Pred | 0.94 | 0.18 | **2.44** | **0.013** |
| Chrysomelidae | 264 | 5 | 2% | 3805 | 71 | Terrestrial | Phyto | Phyto | -0.96 | 0.83 | -1.13 | 0.87 |
| Coccinellidae | 108 | 4 | 5% | 2481 | 9 | Terrestrial | Pred | Pred | 0.63 | 0.24 | -0.12 | 0.51 |
| Cryptophagidae | 68 | 3 | 5% | 428 | 5 | Terrestrial | Fung | Fung | 0.73 | 0.19 | 1.34 | 0.08 |
| Curculionidae | 364 | 8 | 2% | 7453 | 11 | Terrestrial | Phyto | Phyto | -0.45 | 0.68 | -0.40 | 0.66 |
| Dytiscidae | 120 | 36 | 43% | 1531 | 140 | Aquatic | Pred | Pred | 1.29 | 0.10 | 0.97 | 0.17 |
| Elateridae | 251 | 5 | 2% | 3035 | 20 | Terrestrial | Phyto | Phyto | 0.05 | 0.53 | 0.24 | 0.39 |
| Gyrinidae | 25 | 7 | 39% | 215 | 22 | Aquatic | Pred | Pred | **1.87** | **0.05** | 1.06 | 0.16 |
| Haliplidae | 15 | 6 | 67% | 75 | 6 | Aquatic | Phyto | Phyto | **1.80** | **0.05** | 0.87 | 0.21 |
| Hydrophilidae | 62 | 6 | 11% | 265 | 13 | Aquatic | Phyto | Pred | 0.97 | 0.17 | **2.25** | **0.01** |
| Latridiidae | 83 | 3 | 4% | 4216 | 11 | Terrestrial | Fung | Fung | -0.40 | 0.62 | -0.30 | 0.58 |
| Leiodidae | 129 | 5 | 4% | 593 | 19 | Terrestrial | Fung | Fung | -0.25 | 0.58 | -0.47 | 0.67 |
| Scirtidae | 46 | 3 | 7% | 2881 | 9 | Aquatic | Phyto | Phyto | 0.66 | 0.23 | 0.35 | 0.27 |
| Staphylinidae | 972 | 21 | 2% | 7187 | 35 | Terrestrial | Pred | Pred | 0.15 | 0.45 | 1.18 | 0.12 |

Appendix 3: References Used for Determining Traits at Genus and Family Level

Allaby, M. (2014). *A Dictionary of Zoology.* – Oxford University Press, Oxford.

Arango, R.A. (2016). Beetles (Coleoptera) of Peru: a survey of the families. Ptinidae Latreille, 1802. *Journal of the Kansas Entomological Society, 89*(3), 249-252. doi: 10.2317/0022-8567-89.3.249

Balke, M., Wewalka, G., Alarie, Y. & Ribera, I. (2006). Molecular phylogeny of Pacific island Colymbetinae: radiation of New Caledonian and Fijian species (Coleoptera, Dytiscidae). *Zoologica Scripa, 36*(2), 173-200. doi:10.1111/j.1463-6409.2006.00265.x

Ball, O.J.P., Gwinn, K.D., Pless, C.D. & Popay, A.J. (2011). Endophyte isolate and host grass effects on *Chaetocnema pulicaria* (Coleoptera: Chrysomelidae) feeding. (2011). *Journal of Economic Entomology, 104*(2), 665-672. doi:10.1603/EC10262

Beran, F., Pauchet, Y., Kunert, G., Reichelt, M., Wielsch, N., Vogel, H., Reinecke, A., Svatos, A., Mewis, I., Schmid, D., Ramasamy, S., Ulrichs, C., Hansson, B.S., Gershenzon, J. & Heckel, D.G. (2014). *Phyllotreta striolata* flea beetles use host plant defense compounds to create their own glucosinolate-myrosinase system. *Proceedings of the National Academy of Sciences of the United States, 111*(20), 7349-7354. doi:10.1073/pnas.1321781111

Berthiaume, R., Hebert, C. & Cloutier, C. (2012). *Podabrus rugosulus* (Coleoptera: Cantharidae), an opportunist predator of *Mindarus abietinus* (Hemiptera: Aphididae) in Christmas tree plantations. *The Canadian Entomologist, 133*(1), 151-154. doi: 10.4039/Ent133151-1

Bilton, D.T., Hayward, J.W.G., Rocha, J. & Foster, G.N. (2016). Sexual dimorphism and sexual conflict in the diving beetle *Agabus uliginosus* (L.) (Coleoptera: Dytiscidae). *Biological Journal of the Linnean Society, 119*(4), 1089-1095. doi:10.1111/bij.12850

Brown, H.P. (2008). Riffle beetles (Coleoptera: Elmidae). In J. L. Capinera, *Encyclopedia of Entomology.* Retrieved from Gale Virtual Reference Library.

Buss, B.C., Moussallem, M. & Caron, E. (2018). Rediscovery and new subgenus assignment of *Aleochara repetita* Sharp (Coleoptera: Staphylinidae: Aleocharinae). *The Coleopterists Bulletin, 72*(4), 702-706. doi: 10.1649/0010-065X-72.4.702

Capogreco, J.V. (1989). Immature *Lebia viridis* Say (Coleoptera:Carabidae): bionomics, descriptions, and comparisons to other Lebia species. *The Coleopterists Bulletin, 43*(2), 183-194. doi: stable/4008635

Chandra, G., Mandal, S.K., Ghosh, A.K., Das, D., Banerjee, S.S. & Chakraborty, S. (2008). Biocontrol of larval mosquitoes by *Acilius sulcatus* (Coleoptera: Dytiscidae). *BMC Infectious Diseases, 8*(1), 138. doi:10.1186/1471-2334-8-138

Chernov, Y.I., Makarova, O.L., Penev, L.D. & Khruleva, O.A. (2014). Beetles (Insecta, Coleoptera) in the Arctic fauna: communication 1. Faunal composition. *Entomological* *Review, 94*(4), 438-441. doi:10.1134/S0013873814040022

Coyle, D.R., Allred, A.M., Kosola, K.R. & Raffa, K.F. (2010). Altered GAI activity of hybrid aspen has minimal effects on the performance of a polyphagous weevil, *Polydrusus sericeus.* *Entomologia Experimentalis et Applicata, 138*(2), 104-109. doi:10.1111/j.1570-7458.2010.01079.x

Cuthbertson, A.G.S. (2015). Chemical and ecological control methods for *Epitrix* spp. *Global* *Journal of Environmental Science and Management, 1*(1), 95-97. doi: 10.7508/gjesm.2015.01.008

Drees, C., Brandmayr, P., Buse, J., Dieker, P., Gurlich, S., Habel, J., Harry, I., Hardtle, W., Matern, A., Meyer, H., Pizzolotto, R., Quante, M., Schafer, K., Schuldt, A., Taboada, A. & Assmann, T. (2011). Poleward range expansion without a southern contraction in the ground beetle *Agonum viridicupreum* (Coleoptera, Carabidae). *ZooKeys, 100*, 333-352. doi:10.3897/zookeys.100.1535

Drotz, M.K., Brodin, T., Saura, A. & Giles, B.E. (2012). Ecotype differentiation in the face of gene flow within the diving beetle *Agabus bipustulatus* (Linnaeus, 1767) in Northernn Scandinavia. *PLoS ONE, 7*(2), e31381. doi:10.1371/journal.pone.0031381

Du, J., Andreassen, L.D. & Holliday, N.J. (2017). Behavioural responses to dimethyl disulphide by *Aleochara bilineata* and *Aleochara bipustulata.* *Physiological Entomology, 43*(1), 20-29. doi: 10.1111/phen.12221

Dubois, T., Hajek, A.E. & Smith, S. (2002). Methods for rearing the Asian longhorned beetle (Coleoptera:Cerambycidae) on artificial diet. *Annals of the Entomological Society of America, 95*(2), 223-230. doi: 10.1043/0013-8746(2002)095(0223:MFRTAL)2.0.CO2

Elliot, J.M. (2008). The ecology of riffle beetles (Coleoptera: Elmidae). *Freshwater Reviews, 1*(2), 189-203. doi: 10.1608/FRJ-1.2.4

Elshayeb, M. (2006). *Determining food web impacts on experimental aquatic systems from the disposal of oil sands process-affected waste materials*. Msc Thesis, University of Waterloo, Waterloo.

Eyre, D. & Giltrap, N. (2012). *Epitrix* flea beetles: new threats to potato production in Europe. *Pest Management Science, 69*(1), 3-6. doi:10.1002/ps.3423

Fender, K.M. (1973). Ecological notes on *Podabrus* (Coleoptera: Cantharidae). *The Coleopterists Bulletin, 27*(1), 11-17. doi: stable/3999623

Figueroa-Castro, P., Lopez-Martinez, V., Toledo-Hernandez, V.H. & Rifkind, J. (2017). First report of the entomophagous *Enoclerus zonatus* (Coleoptera: Cleridae) associated with stalks of the mezcal maguey in Guerrero, Mexico. *Revista Mexicana de Biodiversidad, 88*(2), 467-470. doi: 10.1016/j.rmb.2017.03.025

Garcia, M., Farinos, G.P., Castanera, P. & Ortego, F. (2012). Digestion, growth and reproductive performance of the zoophytophagous rove beetle *Philonthus quisquiliarius* (Coleoptera: Staphylinidae) fed on animal and plant based diets. *Journal of Insect Physiology, 58*(10), 1334-1342. doi: 10.1016/j.jinsphys.2012.07.007

Gusarov, V.I. (2018). Phylogeny of the family Staphylinidae based molecular data: a review. In Betz, O., Irmler, U. & Klimaszewski, J. (Eds.), *Biology of rove beetles (Staphylinidae): Life history, evolution, ecology and distribution* (pp. 7-25). Springer International Publishing, Switzerland

Heinrich, B. & Vogt, F.D. (1980). Aggregation and foraging behavior of whirligig beetles (Gyrinidae). *Behavioural Ecology and Sociobiology, 7*(3), 179-186. doi:10.1007/BF00299362

Herbst, C., Baier, B., Tolasch, T. & Steidle, J.L.M. (2010). Demonstration of sex pheromones diving beetle *Rhantus suturalis* (MacLeay 1825) (Dytiscidae). *Chemoecology, 21*(3), 19-32. doi:10.1007/s00049-010-0061-3

Hernandez-Juarez, A., Aguirre, L.A., Cerna, E., Landeros, J., Frias, G.A., Flores, M. & Ochoa, Y.M. (2018). Effect of transgenic maize on abundance of the corn flea beetle, *Chaetocnema pulicaria* Melsheimer, as a non-target pest. *Southwestern Entomologist, 43*(4), 841-846. doi: 10.3958/059.043.0403

Hicks, B.J. (1994). Foregut contents of adult *Ilybius* Erichson (Coleoptera: Dytiscidae) from Newfoundland. *The Coleopterists Bulletin, 48*(2), 199-200. ISSN: 0010-065X

Hicks, B.J. & Larson, D.J. (1995). Life history patterns of *Ilybius* Erichson from Newfoundland (Coleoptera: Dytiscidae). *The Coleopterists Bulletin, 49*(3), 281-287. ISSN 0010065X

Hojland, D.H., Nauen, R., Foster, S.P., Williamson, M.S. & Kristensen, M. (2015). Incidence, spread and mechanisms of pyrethroid resistance in European populations of the cabbage stem flea beetle, *Psylliodes chrysocephala* L. (Coleoptera: Chrysomelidae). *PLoS One, 10*(12), e0146045. doi: 10.1371/journal.pone.0146045

Hunting, W.M. (2013). A taxonomic revision of the *Cymindis* *(Pinacodera) limbata* species group (Coleoptera, Carabidae, Lebiini), including description of a new species from Florida, USA. *ZooKeys, 260*, 1-73. doi:10.3897/zookeys.259.2970

Jia, L-P. & Liang, A-P. (2014). An apposition-like compound eye with a layered rhabdom in the small diving beetle *Agabus japonicus* (Coleoptera, Dytiscidae). *Journal of Morphology*, *275*(11), 1273-1283. doi:10.1002/jmor.20300

Karlsson, A.-K.B., Henrikson, B.-I., Harlin, C., Ivarsson, P., Stenson, J.A.E. & Svensson, B.W. (1999). The possible role of volatile secretions as intra- and interspecific alarm signals in *Gyrinus* species. *Oikos, 87*(2), 220-227. doi:10.2307/3546737

Karns, K. & Behrendt, M. (2015). *Lordithon* Thomson, 1859 (Coleoptera: Staphylinidae: Tachyporinae) recorded in Southeastern Ohio, USA, with notes on four rarely collected species including the black Lordithon rove beetle, *Lordithon niger* (Gravenhorst). *The Coleopterists Bulletin, 69(*1), 118-120. doi:10.1649/0010-065X-69.1.118

Kelley, S.T. & Dobler, S. (2011). Comparative analysis of microbial diversity in *Longitarsus* flea beetles (Coleoptera: Chrysomelidae). *Genetica, 139*(5), 541-550. doi:10.1007/s10709-010-9498-0

Keszthelyi, S. (2012). Evaluation of flight phenology and number of generations of the four-spotted sap beetle, *Glischrochilus quadrisignatus* in Europe. Bulletin of Insectology, *65*(1), 9-16. ISSN 1721-8861

Kyneb, A. & Toft, S. (2006). Effects of maternal diet quality on offspring performance in the rove beetle *Tachyporus hypnorum*. *Ecological Entomology, 31*(4), 322-330. doi: 10.1111/j.1365-2311.2006.00775.x

Lawrence, J.F., Hastings, A.M., Dallwitz, M.J., Paine, T.A. & Zurcher, E.J. (2018). *Throscidae.* Elateriformia (Coleoptera). Delta-intkey.com

Leschen, R.A.B. & Beutel, R.G. (2000). Pseudotracheal tubes, larval head, and mycophagy in *Sepedophilus* (Coleoptera: Staphylinidae: Tachyporinae). *Journal of Zoological Systematics and Evolutionary Research, 39*(1-2), 25-35. doi: 10.1046/j.1439-0469.2001.00149.x

Lin, H. & Phelan, P.L. (1991). Identification of food volatiles attractive to *Glischrochilus quadrisignatus* and *Glischrochilus fasciatus* (Coleoptera: Nitidulidae). *Journal of Chemical Ecology, 17*(12), 2469-2480. doi:10.1007/BF00994595

Lloyd, J.E. (2008). Fireflies (Coleoptera: Lampyridae). In J. L. Capinera, *Encyclopedia of Entomology.* Retrieved from Gale Virtual Reference Library.

Lyubarsky, G. & Perkovsky, E. (2011). New species of *Stilbus* (Coleoptera, Clavicornia, Phalacridae) from the Late Eocene Rovno Amber. *Vestnik Zoologii, 45*(2), e-47. doi: 10.2478/v10058-011-0012-7

Majka, C.G. & Langor, D. (2008). The Leiodidae (Coleoptera) of Atlantic Canada: new records, faunal composition, and zoogeography. *ZooKeys, 2*, 357-402. doi: 10.3897/zookeys.2.56

Majka, C.G. & Pollock, D.A. (2006). Understanding saproxylic beetles: new records of Tetratomidae, Melandryidae, Synchroidae, and Scraptiidae from the maritime provinces of Canada (Coleoptera: Tenebrionidae). *Zootaxa, 1248,* 45-68. ISSN 1175-5334

Marshall, S. (2006). *Insects: their natural history and diversity: with a photographic guide to insects of eastern North America*. Firefly Books, New York.

Ming, Q-L & Lewis, S.M. (2010). Pheromone production by male *Tribolium castaneum* (Coleoptera: Tenebrionidae) is influenced by diet quality. *Journal of Economic Entomology, 103(*5), 1915-1919. doi:10.1603/EC10110

Morales-Ramos, J.A., Rojas, M.G., Shapiro-Ilan, D.I. & Tedders, W.L. (2011). Self-selection of two diet components by *Tenebrio molitor* (Coleoptera: Tenebrionidae) larvae and its impact on fitness. *Environmental entomology, 40*(5), 1285-1294. doi: 10.1603/EN10239

Muona, J., Lawrence, J.F. & Slipinski, A. (2010). Throscidae Laporte, 1840. In R.G. Beutel, J.F. Lawrence & R.A.B. Leschen (Eds), *Handbook of Zoology* (pp.69-74). De Gruyter, Berlin.

Odnosum, V. & Litvin, O. (2009). Description of *Mordellistena parvuliformis* larva (Coleoptera, Mordellidae). *Vestnik Zoologii, 43*(6), e-18. doi:10.2478/v10058-009-0023-9

Orfinger, A.B. & Kelly, S.L. (2017). *Tachyporus nitidulus* (Fabricius, 1781) (Coleoptera, Staphylinidae, Tachyporinae): first record from the state of Florida, USA. *Check List, 13*(6), 921-923. doi: 10.15560/13.6.921

Otero, J.C. & Lopez, M.J. (2011). A new species of Cryptophagus herbst (Coleoptera: Cryptophagidae) from the Iberian Peninsula. *The Coleopterist Bulletin, 65*(2), 185-188. ISSN 0010065X

Ortuno, V.M. & Arribas, O. (2018). A revision of the *Cymindis ehlersi* complex (Coleoptera: Carabidae: Lebiinae) with description of a new species and ecological notes. *Zoologischer Anzeiger, 276,* 1-14. doi:10.1016/j.jcz.2018.05.002

Otto, R.L. (2017). Beetles of Peru: a survey of the families. Eucnemidae Eschscholtz, 1829. *Revista Peruana de Biologia, 24*(1), 11-24. doi: 10.15381/rpb.v24i1.13107

Pan, P., Yang, X., Siegfried, B.D. & Zhou, X. (2015). A comprehensive selection of reference genes for RT-qPCR analysis in a predatory lady beetle, *Hippodamia convergens* (Coleoptera: Coccinellidae). *PLoS ONE, 10*(4), e0125868. doi: 10.1371/journal.pone.0125868

Peck, S.B. & Newton, A.F. (2017). An annotated catalog of the Leiodidae (Coleoptera) of the Nearctic region (Continental North America North of Mexico). *The Coleopterists Bulletin, 71*(2), 211-258. doi: 10.1649/0010-065X-71.2.211

Pinski, R.A., Mattson, W.J. & Raffa, K.F. (2005). Host breadth and ovipositional behavior of adult *Polydrusus sericeus* and *Phyllobius oblongus* (Coleoptera: Curculionidae), nonindigenous inhabitants of northern hardwood forests. *Environmental Entomology, 34*(1), 148-157. doi:10.1603/0046-225X-34.1148

Rees, D.P. & Rangsi, V. (2004). *Insects of stored products.* CSIRO Publishing, Victoria.

Rodrguez-del-Bosque, L.A. (2013). Feeding and survival of *Oncideres pustulata* (Coleoptera: Cerambycidae) adults on *Acacia farnesiana* and *Leucaena leucocephala* (Fabaceae). – *Southwest Entomologist, 38*(3), 487-498. doi: 10.3958/059.038.0311

“Scarab beetle”. (2020). In The Editors of Encyclopedia Britannica (Ed), *Encyclopaedia Britannica.* Retrieved from Britannica.com.

Selnekovic, D. & Kodada, J. (2019). Taxonomic revision of *Mordellistena hirtipes* species complex with new distribution records (Insecta, Coleoptera, Mordellidae). *ZooKeys, 854*, 89-118. doi:10.3897/zookeys.854.32299

Siposova, D., Ciamporova-Zatovicova, Z. & Ciampor Jr, F. (2017). Development of microsatellite loci for two *Agabus* diving beetle species from the pooled DNA and testing their utility in mountain lake populations. *Limnologica, 67*, 7-19. doi:10.1016/j.limno.2017.09.002

Staniec, B., Zagaja, M., Pietrykowska-Tudruj, E. & Wagner, G.K. (2018). Comparative larval ultramorphology of some myrmecophilous Aleocharinae (Coleoptera, Staphylinidae), with a first description of the larvae of *Amidobia talpa* (Heer O, 1841) and *Oxypoda haemorrhoa* (Mannerheim C.G., 1830), associated with the Formica rufa species group. *ZooKeys, 808*, 93-114. doi: 10.3897/zookeys.808.29818

Steury, B.W., Steiner Jr., W.E. & Shockley, F.W. (2018). The soldier beetles and false soldier beetles (Coleoptera: Cantharidae and Omethidae) of the George Washington Memorial Parkway. *The Maryland Entomologist, 7*(2), 11-27

Svensson, B.W. (1992). Changes in occupancy, niche breadth and abundance of three *Gyrinus* species as their respective range limits are approached. *Oikos, 63*(1), 147-156. doi:10.2307/3545524

Tilden, J.W. (1950). The feeding of *Podabrus pruinosus* LeConte (Cantharidae). *The Coleopterists Bulletin, 4*(6), 92. doi: stable/3998496

Tree of Life Web Project. (2011). *Scirtidae. Marsh beetles.* Tree of Life Web Project. <http://tolweb.org/Scirtidae/9613>

Triplehorn, C. A. (2008). Darkling beetles (Coleoptera: Tenebrionidae). In J. L. Capinera, *Encyclopedia of Entomology.* Retrieved from Gale Virtual Reference Library.

Urban, J. (2011). Occurrence, bionomics and harmfulness of *Crepidodera aurea* (Geoffr.) (Coleoptera, Alticidae). *Acta Universitatis Agriculturae et Silviculturae Mendelianae Brunensis, 59*(5), 279-308. doi: 10.11118/actaun201159050279

Vahtera, V., Muona, J., Linna, A. & Saaksjarvi, I.E. (2015). Nine genera of Eucnemidae (Coleoptera) new to Peru, with a key to Peruvian genera. *Biodiversity Data Journal,3*, e4493. doi: 10.3897/BDJ.3.e4493

Vondel, B.J.V. & Alarie, Y. (2016). A new species of *Haliplus* Latreille, 1802 (Coleoptera: Adephaga: Haliplidae) from Canada. *The Coleopterists Bulletin, 70*(4), 801-804. doi:10.1649/0010-065X-70.4.801

Walczynska, A. (2010). Is wood safe for its inhabitants? *Bulletin of Entomological Research, 100*(4), 461-465. doi: 10.1017/S0007485309990514

Webster, R.P., Sweeney, J.D. & DeMerchant, I. (2012). New Coleoptera records from New Brunswick, Canada: Mordellidae and Ripiphoridae. *ZooKeys, 179*, 243-256. doi: 10.3897/zookeys.179.2583

Webster, R.P., Sweeney, J.D. & DeMerchant, I. (2012). New Coleoptera records from New Brunswick, Canada: Trogossitidae, Cleridae, and Melyridae, with an addition to the fauna of Nova Scotia. *ZooKeys, 179*, 141-156. doi: 10.3897/zookeys.179.2585

“Weevils”. (2014). In K. L. Lerner & B. W. Lerner, *The Gale Encyclopedia of Science.* Retrieved from Gale Virtual Reference Library.

Yavorskaya, M.I., Leschen, R.A.B., Polilov, A.A. & Beutel, R.G.(2014). Unique rostrate larvae and basidiomycophagy in the beetle family Corylophidae. *Arthropod Structure & Development, 43*(2), 153-162. doi: 10.1016/j.asd.2013.11.001

Yee, D.A. (2014). Ecology, systematics, and the natural history of predaceous diving beetles (Coleoptera: Dytiscidae). Springer, Dordrecht.

Zhang, S.-Q., Che, L.-H., Li, Y., Liang, D., Pang, H., Ślipiński, S.A. & Zhang, P. (2018). Evolutionary history of Coleoptera revealed by extensive sampling of genes and species. *Nature Communications, 9*(1), 1-11. doi: 10.1038/s41467-017-02644-4

Zhou, J., Ross, D.W., Niwa, C.G. (2001). Kairomonal response of *Thanasimus undatulus*, *Enoclerus sphegeus* (Coleoptera: Cleridae), and *Temnochila chlorodia* (Coleoptera: Trogositidae) to bark beetle semiochemicals in Eastern Oregon. *Environmental Entomology, 30*(6), 993-998. doi: 10.1603/0046-225X-30.6.993

Zimmer, C.T., Muller, A., Heimbach, U. & Nauen, R. (2014). Target-site resistance to pyrethroid insecticides in German populations of the cabbage stem flea beetle, *Psylliodes chrysocephala* L. (Coleoptera: Chrysomelidae). *Pesticide Biochemistry and Physiology, 108*, 1-7. doi: 10.1016/j.pestbp.2013.11.005
